# Functional role of dimerization and CP190 interacting domains of CTCF protein in *Drosophila melanogaster*

**DOI:** 10.1186/s12915-015-0168-7

**Published:** 2015-08-07

**Authors:** Artem Bonchuk, Oksana Maksimenko, Olga Kyrchanova, Tatyana Ivlieva, Vladic Mogila, Girish Deshpande, Daniel Wolle, Paul Schedl, Pavel Georgiev

**Affiliations:** Department of the Control of Genetic Processes, Institute of Gene Biology, Russian Academy of Sciences, Moscow, Russia; Laboratory of Gene Expression Regulation in Development, Institute of Gene Biology, Russian Academy of Sciences, Moscow, Russia; Department of Molecular Biology, Princeton University, Princeton, NJ USA; Department of Biology, Nikolaev V.A. Sukhomlinsky National University, Nikolaev, Ukraine

**Keywords:** Bithorax complex, BTB domain, Chromatin insulator, Embryo development, Transcription

## Abstract

**Background:**

Insulators play a central role in gene regulation, chromosomal architecture and genome function in higher eukaryotes. To learn more about how insulators carry out their diverse functions, we have begun an analysis of the *Drosophila* CTCF (dCTCF). CTCF is one of the few insulator proteins known to be conserved from flies to man.

**Results:**

In the studies reported here we have focused on the identification and characterization of two dCTCF protein interaction modules. The first mediates dCTCF multimerization, while the second mediates dCTCF–CP190 interactions. The multimerization domain maps in the N-terminus of the dCTCF protein and likely mediates the formation of tetrameric complexes. The CP190 interaction module encompasses a sequence ~200 amino acids long that spans the C-terminal and mediates interactions with the N-terminal BTB domain of the CP190 protein. Transgene rescue experiments showed that a dCTCF protein lacking sequences critical for CP190 interactions was almost as effective as wild type in rescuing the phenotypic effects of a *dCTCF* null allele. The mutation did, however, affect CP190 recruitment to specific *Drosophila* insulator elements and had a modest effect on dCTCF chromatin association. A protein lacking the N-terminal dCTCF multimerization domain incompletely rescued the zygotic and maternal effect lethality of the null and did not rescue the defects in *Abd-B* regulation evident in surviving adult *dCTCF* mutant flies. Finally, we show that elimination of maternally contributed *dCTCF* at the onset of embryogenesis has quite different effects on development and *Abd-B* regulation than is observed when the homozygous mutant animals develop in the presence of maternally derived *dCTCF* activity.

**Conclusions:**

Our results indicate that dCTCF–CP190 interactions are less critical for the in vivo functions of the dCTCF protein than the N-terminal dCTCF–dCTCF interaction domain. We also show that the phenotypic consequences of *dCTCF* mutations differ depending upon when and how *dCTCF* activity is lost.

**Electronic supplementary material:**

The online version of this article (doi:10.1186/s12915-015-0168-7) contains supplementary material, which is available to authorized users.

## Background

The chromatin fiber in the chromosomes of multicellular animals is organized into a hierarchical set of topologically independent domains [1–6]. This hierarchical loop domain organization is critical for the proper utilization and propagation of the genetic information encoded by the chromosome and it is intimately involved in such processes as gene regulation, replication, recombination, repair, and mitosis. A special set of architectural elements, called boundaries or insulators, are responsible for both subdividing the chromatin fiber into discrete domains and determining their hierarchical organization. These architectural elements have a seemingly contradictory set of functions. When placed between enhancers/silencers and their target genes, they block regulatory interactions [1, 7–11]. However, insulators also have the ability to promote regulatory interactions between enhancers/silencers and their target genes. One mechanism depends upon insulator pairing interactions that connect distant chromosomal segments together at the base of a topological loop [12, 13]. When the regulatory elements in the loop are properly oriented, these insulator–insulator pairing interactions can place distant enhancers/silencers in close proximity to target genes. Additionally, there is suggestive evidence that insulators can have an even more direct role in mediating enhancer–promoter interactions [14–21].

Nearly a dozen DNA binding proteins that have insulator/architectural activity have been discovered. Of these, the most broadly conserved protein is the CCCTC-binding factor, CTCF [22]. Except for a few lineages that have apparently lost the CTCF gene, it is a characteristic feature of bilaterian organisms [23–25]. CTCF was initially identified as a transcription factor that can both repress and activate transcription [26, 27]. Subsequent experiments in flies and vertebrates showed that CTCF also has enhancer-blocking activity and to date it is the only mammalian protein has been shown to have this insulator activity [28–30]. In addition to its roles in gene regulation, a growing body of evidence points to an architectural function. For example, in vertebrates CTCF mediates specific long-distance interactions between insulators and regulatory elements located at megabase distances and even on different chromosomes [31–34]. Similarly, *Drosophila* dCTCF can support pairing-dependent insulator bypass in transgene assays when two sets of multimerized binding sites for the protein are arranged in the appropriate orientation [15, 35].

Like the CTCF proteins of vertebrates, *Drosophila* CTCF contains 11 C2H2 zinc fingers flanked by N-terminal domains (NTDs) and C-terminal domains (CTDs) [36]. The dCTCF zinc fingers show significant homology with their vertebrate counterparts, and this is reflected in the similar sequence recognition properties of the vertebrate and fly proteins [37]. In contrast, the NTDs and CTDs are not well conserved and there are reasons to think that the differences between the NTDs and CTDs of vertebrate and fly proteins have mechanistic implications. The insulator/architectural activities of vertebrate CTCF depend at least in part upon its ability to recruit the cohesin complex to specific chromosomal sites [38–41]. Cohesin knockdowns were shown to impair both the insulator (enhancer-blocking) and architectural (long-distance interactions) functions of CTCF [31, 42, 43]. The vertebrate cohesin complex consists of four proteins: Smc1, Smc3, Scc1, and SA/STAG [44]. Smc1 and Smc3 form a ring in the presence of ATP, and this ring is stabilized by the binding of Scc1 and SA/STAG. Vertebrate CTCF is thought to recruit cohesins to specific chromosomal sites by interacting directly with the SA/STAG subunit of cohesin complex. Xiao et al. [45] have shown that sequences in the C-terminal tail of CTCF are responsible for specific interactions with SA/STAG, and that insulator activity and cohesin recruitment are disrupted when this region of the CTCF protein is mutated.

dCTCF differs from its vertebrate counterpart in that it does not appear to co-localize with cohesins [44, 46]. This finding has led to the idea that other proteins might fulfill the long-distance linking function envisioned for cohesins. One plausible candidate is CP190 [47]. It was originally identified as a microtubule binding protein that associates with the centrosome during mitosis [48, 49]. However, subsequent studies argued against a centrosome or mitotic function and instead pointed to a role in some aspect of nuclear architecture or chromosome structure [50, 51]. Support for this idea came from the discovery that CP190 is required for the enhancer-blocking activity of the *gypsy* Su(Hw) insulator and is a component both of this transposon insulator and of endogenous Su(Hw) insulators [52, 53]. The connection to chromosome architecture was further supported by studies showing that CP190 localizes to many dCTCF sites and can be co-immunoprecipitated with dCTCF [54–57].

In the studies reported here we have examined the functioning dCTCF protein in more detail. Using a combination of biochemical and genetic approaches we have identified the CP190 interaction domain. We also uncovered a dCTCF dimerization/multimerization domain that, like CP190, could potentially mediate interactions between distant DNA sequences containing dCTCF in vivo. In the course of this analysis we have re-examined the effects of dCTCF null mutations, and tested whether dCTCF proteins lacking the CP190 interaction domain or the dCTCF dimerization/multimerization domain can rescue the null mutation.

## Results

### dCTCF contains an N-terminal dimerization domain

**Fig. 1:**
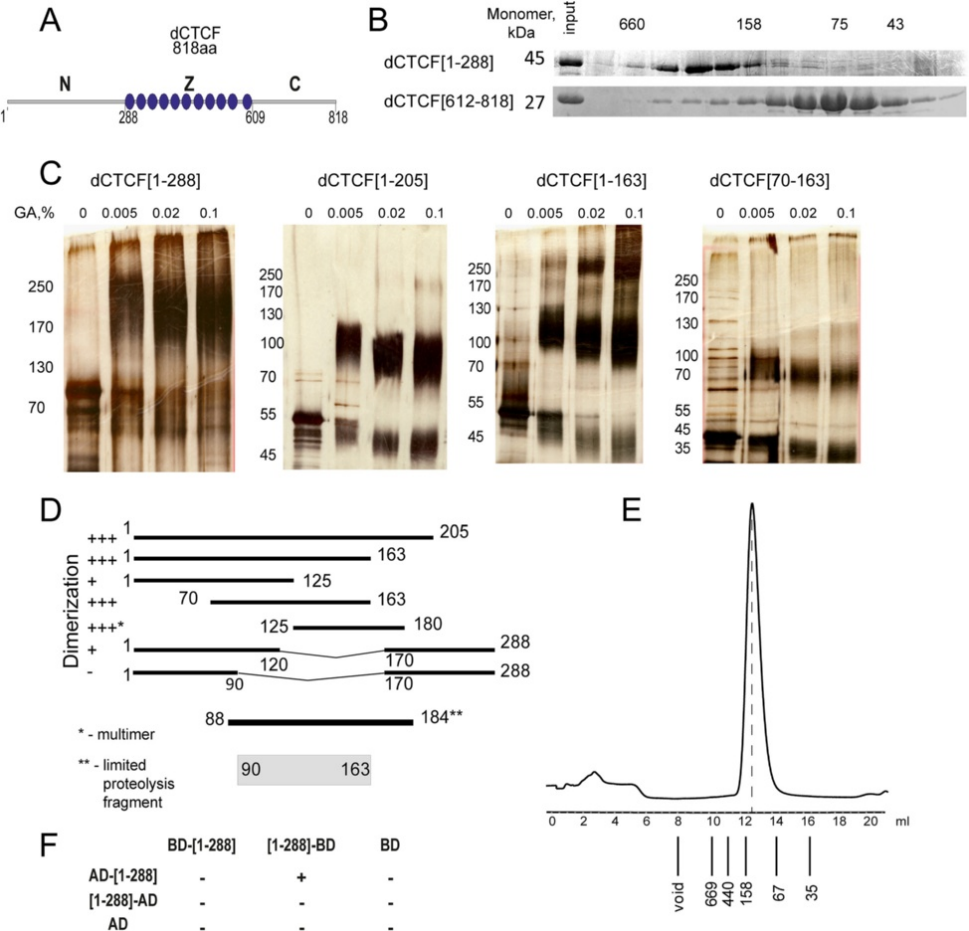
**a** Domain structure of the dCTCF protein. **b** Sephacryl S200 size-exclusion chromatography of dCTCF terminal domains. (N-terminal domain is thioredoxin-tagged.) Positions of molecular weight markers are shown. **c** Cross-linking of dCTCF N-terminal thioredoxin-tagged deletion derivatives using increasing concentrations of glutaraldehyde (*GA*). Proteins were separated in a 5–12 % gradient SDS-PAGE gels and visualized with silver-staining. **d** Summary of the results from chemical cross-linking mapping experiments and limited proteolysis of the dCTCF–NTD multimerization domain. For further experiments see Additional file 2: Figure S2. **e** Superdex 200 size-exclusion chromatography of dCTCF 1–163 amino acids without thioredoxin. **f** Analysis of dCTCF protein N-terminal dimerization using yeast two-hybrid assay. Relative N- or C- terminal position of AD/BD is shown. *AD* GAL4 activation domain, *BD* GAL4 DNA binding domain

The 11 zinc fingers of the CTCF proteins are highly conserved in bilaterian phyla [24, 25] (see schematic in Fig. 1a and Additional file 1: Figure S1A). In contrast, the NTDs and CTDs were poorly conserved and there was little sequence similarity even between proteins from different dipteran families (Additional file 1: Figure S1A). Sequence alignment of CTCF proteins from species within the *Drosophila* genus revealed much more extensive homology in the NTDs and CTDs, including several very well conserved sequence blocks (Additional file 1: Figure S1B). A plausible hypothesis is that these conserved sequences may serve as protein interaction modules that are important for dCTCF activities.

One of the interactions that could be mediated by these modules was the dimerization or multimerization of the dCTCF protein. This possibility was suggested by insulator bypass experiments in which pairs of multimerized and appropriately oriented CTCF binding sites could mediate long-distance regulatory interactions [15, 16]. Additionally, studies on vertebrate CTCF have suggested that it can form dimers [58, 59]. To test for the presence of homo-dimerization/multimerization modules in the N- or C-terminus of the dCTCF protein, we fractionated bacterially expressed thioredoxin-fused NTD or CTD proteins by size-exclusion chromatography. As shown in Fig. 1b, the thioredoxin NTD fusion 1–288 had a hydrodynamic molecular mass significantly larger (~250 kD) than that predicted for the monomer (45 kD). Similar results were obtained for the CTD 612–818 protein (Fig. 1b).

The presence of these larger complexes could be explained by either a module-dependent multimer formation or by the presence of intrinsically disordered regions that lead to non-specific protein aggregation. To distinguish between these possibilities we used glutaraldehyde cross-linking to probe for complex formation. In the case of the CTD 612–818 protein, glutaraldehyde cross-linking was quite inefficient, suggesting that it likely forms non-specific aggregates (Additional file 2: Figure S2A). On the other hand, consistent with the results of the size-exclusion chromatography, cross-linking of the NTD 1–288 protein gave a high yield of a multimeric band of ~200 kD (Fig. 1c).

To further pinpoint the interaction module, we generated three C-terminal deletions (see Fig. 1d). The smallest deletion, NTD 1–205, gave a cross-linked band of ~100 kD. The largest C-terminal deletion, NTD 1–125, also gave a cross-linked product; however, the yield was quite low compared to the NTD 1–205 protein (compare Additional file 2: Figure S2A to Fig. 1c). This suggests that key interaction sequences were located between amino acids 125 and 205. This suggestion was supported by NTD 1–163, which was much more efficiently cross-linked than NTD 1–125 (Fig. 1c). Though NTD 1–163 gave a prominent cross-linked band at the approximate size expected for the tetramer (~120 kD), there was also a ladder of larger bands. This ladder was likely due, at least in part, to the presence of the thioredoxin moiety in the fusion protein, as only two cross-linked bands were observed when thioredoxin was removed (Additional file 2: Figure S2A). Taken together, these findings map the N-terminal dCTCF:dCTCF multimerization module to sequences spanning the region between amino acids 125 and 163 and suggest that this module likely mediates the formation of dimers or possibly tetrameric complexes*.* Further support for the formation of multimeric complexes (tetrameric or an even larger) came from size-exclusion chromatography of the NTD 1–163 protein (lacking the thioredoxin moiety), which gave a predicted mass of 120 kD (Fig. 1e). However, it is also possible that disordered regions of the protein retard complex mobility during size-exclusion chromatography.

Several additional lines of evidence localized the dCTCF multimerization module to this region of the NTD. First, two internal deletions (Fig. 1d) that lacked sequences from this interval failed to cross-link efficiently (Additional file 2: Figure S2A). Second, two terminally truncated proteins, NTD 125–180 and NTD 70–163 (Fig. 1d), that contained this part of the NTD were cross-linked efficiently (Additional file 2: Figure S2A). Third, protease digestion indicated that the region containing the interaction module had an ordered structure. We subjected the thioredoxin NTD 1–205 fusion protein to limited proteinase K or trypsin digestion and then analyzed the resulting protease-resistant products by matrix-assisted laser desorption/ionization time-of-flight (MALDI-TOF) mass spectrometry (Additional file 2: Figure S2B; Additional file 3: Table S1). Both proteases generated two resistant-to-digestion products. One corresponded to thioredoxin, while the other to a dCTCF NTD peptide extending from 84 to 188.

To independently demonstrate that the NTD contains a dCTCF multimerization module we used two different in vivo assays. The first was a yeast two-hybrid assay (Fig. 1f). Sequences encoding the NTD 1–288 amino acids were fused in-frame to the yeast GAL4 DNA binding domain (BD) and activation domain (AD). Because steric hindrance can interfere with transcriptional activation in the two-hybrid system, the NTD 1–288 sequence was placed at both the N-terminus (NTD-AD and NTD-BD) and the C-terminus (AD-NTD and BD-NTD) of the fusion protein. Fig. 1f shows that activation was observed in only one configuration, NTD-BD and AD-NTD. Similar results were obtained when the NTD was tested with a full-length dCTCF protein (not shown).

**Fig. 2:**
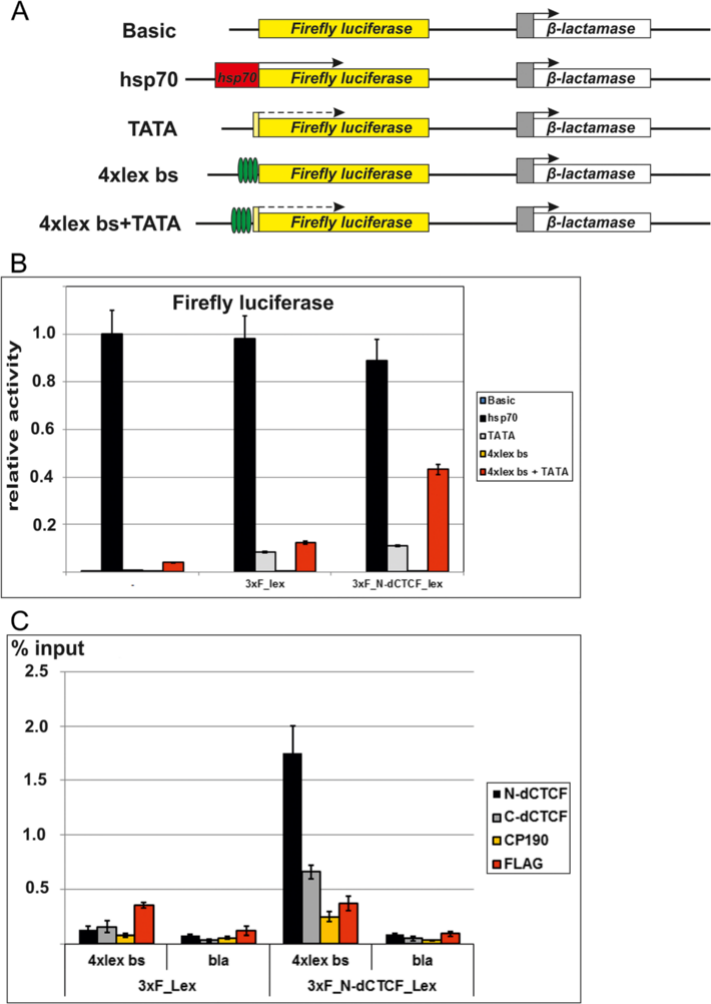
**a** Schematic drawing of luciferase reporter constructs. **b** Firefly luciferase expression from the five reporters shown in *a* when co-transfected with empty vector, with a vector encoding a 3xFLAG-tagged-(nuclear localization signal)-LexA fusion protein, or with a vector encoding the 3xFLAG-tagged N-terminal dCTCF-(nuclear localization signal)-LexA fusion protein. A plasmid encoding the Renilla luciferase under the control of the actin promoter was used to correct for variations in transfection efficiency, and expression of the firefly luciferase was normalized in each case to Renilla luciferase. Each transfection experiment was performed in three independent biological replicates and each lysate was measured in four technical replicates. Error bars show standard deviations of measurements of all summarized replicates. **c** Chromatin immunoprecipitation of S2 cells co-transfected with the 4xLesA TATA-box reporter or the basic promoterless reporter and either of two fusion protein expression constructs, the 3xFLAG-tagged (nuclear localization signal) LexA construct or the 3xFLAG-tagged-N-terminal dCTCF-(nuclear localization signal)-LexA construct. Fixed and processed S2 chromatin samples were immunoprecipitated with antibodies directed against (as indicated) the dCTCF N-terminus, the dCTCF C-terminus, CP190, or FLAG, and then assayed for the presence of sequences corresponding to the 4xLexA TATA reporter or the basic reporter constructs as indicated. Each chromatin immunoprecipitation experiment was performed in three independent biological replicates. Error bars show standard deviations of summarized biological replicates after quadruplicate PCR measurements in each experiment. The results are presented as a percentage of input DNA. *Basic* no promoter, *bla* basic promoterless reporter, *Hsp70* firefly luciferase with an hsp70 promoter, *TATA* firefly luciferase with a minimal TATA-box promoter, *4xlex bs*, firefly luciferase with four copies of the LexA recognition sequence, *4xlex bs + TATA*, firefly luciferase with four copies of the LexA recognition sequence linked to a minimal TATA-box promoter

In the second assay, we ectopically expressed a 3xFLAG-tagged fusion protein consisting of the N-terminal 302 amino acids of dCTCF, a nuclear localization signal and the bacterial LexA DNA BD in *Drosophila* S2 tissue culture cells. The S2 cells were co-transfected with a plasmid encoding the firefly luciferase protein, whose expression is dependent upon a minimal TATA-box promoter and upstream 4xLexA binding sites (Fig. 2a). Measurements of luciferase activity relative to a Renilla luciferase co-transfection control indicated that the 3xFLAG-N-terminal dCTCF-LexA fusion protein weakly activated firefly luciferase expression from the 4xLexA-TATA reporter (Fig. 2b). By contrast, no activation was observed for a luciferase reporter that lacked the 4xLexA binding sites or when the 3xFLAG-tagged fusion protein had the LexA DNA BD but not the dCTCF N-terminal domain. The chromatin immunoprecipitation (ChIP) experiments in Fig. 2c show that when the N-terminal dCTCF fusion protein was tethered to the 4xLexA-TATA reporter via the LexA BD, it could interact with and recruit endogenous full-length dCTCF. Because CP190 antibodies were also able to immunoprecipitate the 4xLexA-TATA reporter, it would appear that the full-length dCTCF protein could in turn recruit CP190 to the 4xLexA-TATA reporter (via the CTD of the full-length dCTCF protein: see below).

### The BTB^CP190^ dimer interacts with the C-terminal domain of dCTCF

**Fig. 3:**
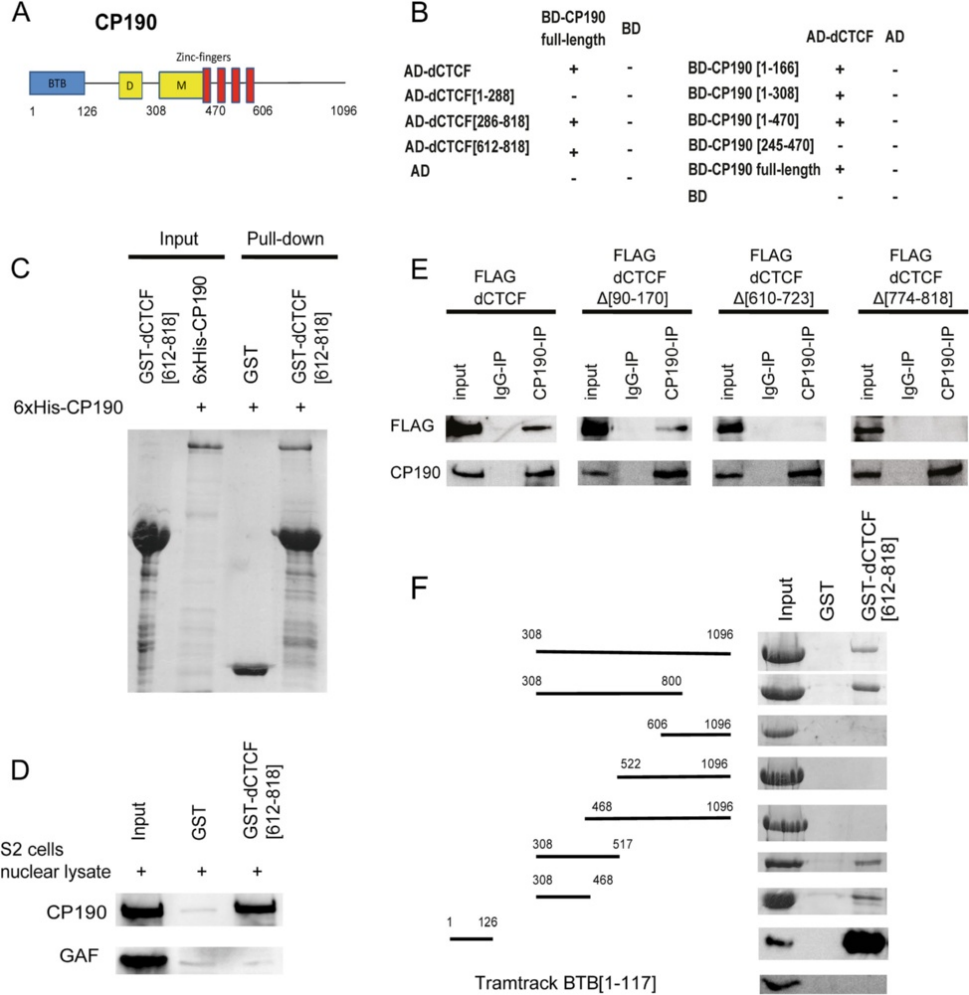
**a** Domain structure of the *Drosophila* CP190 protein. **b** Mapping dCTCF and CP190 interaction modules using the yeast two-hybrid assay. **c** Analysis of interactions between purified recombinant GST-dCTCF-CTD and 6xHis-CP190 by GST-pull-down assay. GST-dCTCF-CTD bound to glutathione agarose beads was incubated with bacterially expressed 6xHis-CP190. After successive washes, the GST-dCTCF-CTD protein was eluted from the beads with excess glutathione. **d** Analysis of interactions between recombinant GST-dCTCF-CTD and CP190 from *Drosophila* S2 cells nuclear lysate by GST-pull-down assay. An S2 nuclear extract was incubated with recombinant GST-dCTCF-CTD bound to glutathione agarose beads. After washing and elution with excess glutathione, CP190 and GAF association was assayed by western blotting. **e** Immunoprecipitation of FLAG-tagged dCTCF full-length and deletion mutants with CP190 antibodies. **f** Mapping of CTCF-interaction region within CP190 protein using GST-pull-down assay. *AD* activating domain, *BD* binding domain, *S2* Schneider 2 cells

CP190 has an N-terminal BTB-POZ (BTB for BR-C, ttk and bab and POZ for Pox virus and Zinc finger) protein–protein interaction domain, which is followed by an aspartic acid-rich D domain, a microtubule targeting domain, four C2H2 zinc fingers (which bind non-specifically to DNA), and finally a glutamic acid rich C terminal domain (Fig. 3a). Previously it was shown that the CP190 protein interacts with dCTCF [54, 55]. While interacting modules in the two proteins were not identified, it was found that the BTB domain is required for the binding of CP190 to chromatin [60].

To identify modules in dCTCF that mediate CP190 interaction, we first used the yeast two-hybrid assay. dCTCF was subdivided into the NTD, the zinc fingers plus the CTD, and the CTD alone and each was fused to the GAL4 activation domain. The NTD failed to interact, while the full-length protein, the zinc fingers plus the CTD, and the CTD alone gave transcriptional activation when combined with full-length CP190 (Fig. 3b). The localization of the CP190 interaction module in dCTCF to the CTD was confirmed by GST pull-down experiments. A GST-fusion protein containing the dCTCF-CTD 612–818 domain was found to pull down both bacterially expressed CP190 and CP190 in *Drosophila* S2 cell nuclear extracts (Fig. 3c,d).

To further pinpoint the sequences within the 612–818 CTD that are important for contacting CP190, we expressed two different FLAG-tagged C-terminal deletions, dCTCFΔ610-723 and dCTCFΔ774-818, in *Drosophila* S2 cells. Figure 3e shows that FLAG-tagged wild-type dCTCF and a control N-terminal deletion could be precipitated by CP190 antibodies from the S2 extracts. In contrast, neither of the smaller dCTCF-CTD deletions was precipitated by CP190 antibodies from S2 cells. Taken together, these findings indicate that an apparently rather large sequence is required to mediate a dCTCF–CP190 association that is stable in S2 nuclear extracts.

We used a similar strategy to localize the region in the CP190 protein that mediates interactions with dCTCF. For the yeast two-hybrid experiments, full-length dCTCF was fused to the GAL4 activation domain, while different sub-fragments from CP190 were fused to the GAL4 DNA BD. These experiments map a dCTCF interaction module to the CP190 BTB domain (Fig. 3b). This was confirmed by GST pull-down experiments (Fig. 3f) which showed strong protein–protein interactions between the CP190 BTB domain (1–126) and dCTCF. In addition, weak interactions were detected between the dCTCF-CTD and GST–CP190 fusions spanning the microtubule interaction domain (see CP190 308–517 and 308–468 in Fig. 3f).

**Fig. 4:**
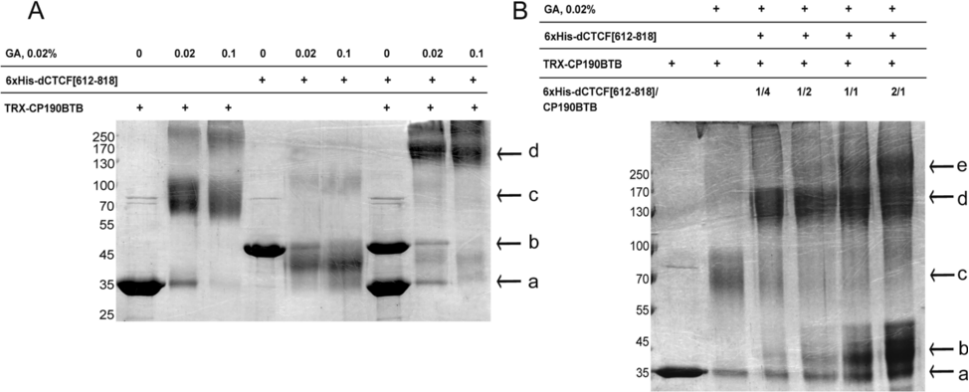
**a** Analysis of complexes between dCTCF-CTD and thioredoxin-tagged CP190-BTB mixed at a molar ratio of 1:2 using the chemical cross-linking reagent glutaraldehyde. Proteins were visualized by Coomassie staining. *a* indicates position of CP190 BTB-domain monomer, *b* position of dCTCF-CTD, *c* dimer of CP190 BTB, *d* complex between CP190 BTB dimer and dCTCF-CTD. **b** Analysis of stoichiometry of interaction between dCTCF-CTD and CP190-BTB mixed in different molar ratios, and cross-linked with 0.2 % glutaraldehyde after 1 h incubation, visualized by silver-staining. *a* indicates position of CP190 BTB-domain monomer, *b* position of CTCF-CTD, *c* dimer of CP190 BTB, *d* complex between CTCF-CTD and CP190 BTB in molar ratio 1:2, and *e* higher order complex between CTCF-CTD and CP190 BTB with unknown stoichiometry. *GA* glutaraldehyde

We have previously shown that the CP190 BTB domain exists as a stable homodimer [61]. This observation raised the possibility that a CP190 dimer could simultaneously bind two dCTCF proteins, linking them together in the same manner that the Bcl6 BTB dimer is thought to bring together two SMRT co-repressors [62]. However, glutaraldehyde cross-linking experiments argue that the predominant complex consists of a BTB^CP190^ dimer linked to a single CTD protein. Figure 4a shows that the CP190 BTB domain alone formed a stable dimer that could be readily captured by glutaraldehyde cross-linking. When the BTB domain was present in a twofold excess over the dCTCF–CTD 612–818 protein, the cross-linked BTB^CP190^ dimer disappeared and was replaced by a band migrating with an apparent molecular weight of ~130 kD. While this cross-linked complex migrated more slowly than we would have predicted, we interpret it to be a 2xBTB^CP190^:CTD 612–818 trimer based on the stoichiometry of the two proteins. This conclusion was supported by cross-linking experiments in the presence of increasing amounts of the CTD protein (Fig. 4b). At a CTD to BTB ratio of 1:4 and 1:2, the predominant cross-linked species was the ~130 kD 2xBTB^CP190^:CTD trimer, whereas there was little if any of the BTB^CP190^ dimer. Only at ratios of 1:1 or 2:1 did we observe a larger species that could correspond to the BTB^CP190^ dimer linked to two CTD proteins or to some other more complex structure(s). However, under these conditions a significant fraction of the CTD protein appeared to be free monomer, and this would also argue that the preferred configuration is a 2xBTB^CP190^:CTD heterotrimer.

### Rescuing a dCTCF null allele

If the NTD and CTD interaction modules are critical for dCTCF function, then dCTCF proteins that lack these modules should be defective. As a prelude to assaying the activity of NTD and CTD mutant proteins in vivo, we determined whether *dCTCF* mutant flies could be rescued by a transgene expressing the wild-type dCTCF protein. Several putative *dCTCF* null alleles have been reported [54, 55, 63]. Flies homozygous for these mutations (*dCTCF*^*30.6*^, *dCTCF*^*Y+1*^, *dCTCF*^*Y+2*^, *dCTCF*^*30*^) mainly died during larvae–pupae stages. For the wild-type rescue construct we generated P-element transformants of a hybrid fusion gene that expresses the *dCTCF* cDNA under the control of the ubiquitously expressed *hsp83* promoter [64, 65]. To identify the transgene proteins, a sequence encoding a 3xFLAG epitope was introduced at the beginning of the dCTCF open reading frame. Five independent transgene inserts were recovered on the first and second chromosomes; however, none of these transgenes rescued the lethal effects of the four *dCTCF* alleles. One reason why the transgenes were unable to complement the four *dCTCF* alleles is that they did not express as much protein as the endogenous *dCTCF* gene. An alternative possibility is that there were additional lethal lesions on the chromosomes carrying these particular *dCTCF* mutations.

**Fig. 5:**
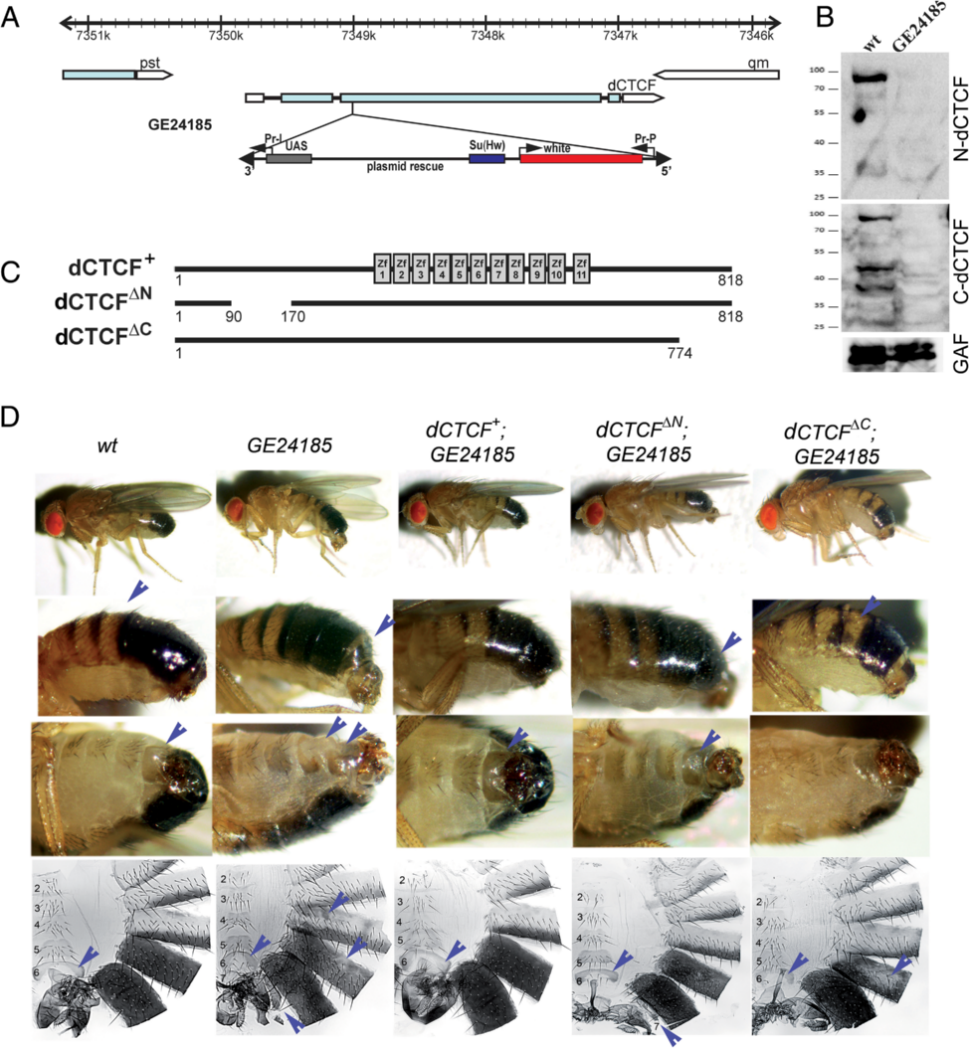
**a** Schematic diagram showing the *GE24185* transposon insertion into the *dCTCF* gene. **b** Western blots of protein extracts prepared from wild-type and homozygous *GE24185* mutant flies. **c** Schematic representation of dCTCF constructs used to rescue the *GE24185* mutation. **d** Abdomen and cuticle preparations (bottom row) of wild-type and homozygous *GE24185* mutant flies in the absence or presence of the *hsp83:dCTCF* transgenes as indicated. Arrows in *GE24185* and *dCTCF* ^*ΔN*^;*GE21485* indicate the presence of a rudimentary A7 tergite and hairs on the A6 sternite. Arrows in *dCTCF* ^*ΔC*^;*GE24185* indicate an A5 to A4 transformation of the tergite. *wt* wild type, *A4-A7* abdominal segments 4-7

A fifth predicted *dCTCF* null allele, *GE24185*, has been described [55]. The viability of adults homozygous for the *GE24185* mutation is reduced by a third or more, while F2 flies do not survive. The *GE24185* mutation was generated by insertion of an EP^S^ transposon in reverse orientation into the third exon of the *dCTCF* gene (Fig. 5a). The EP^S^ transposon contains an *hsp70* minimal promoter that drives transcription in the opposite orientation to the *dCTCF* gene [66]. The promoter is under control of a GAL4-responsive enhancer. As would be expected from its insertion site, the *GE24185* disrupts expression of the dCTCF protein. Extracts prepared from F1 adults homozygous for the *GE24185* mutation showed no dCTCF-specific bands when probed with antibodies directed against N-terminal or C-terminal regions of the dCTCF protein (Fig. 5b). Unlike the other *dCTCF* alleles, we found that two copies of the *hsp83-dCTCF*^*+*^ transgene rescued the F1 and F2 lethal phenotypes of the *GE24185* mutation*.*

To confirm these findings, we generated two imprecise excisions, *GEx52* and *GEx56*, by introducing the P transposase. As indicated in Additional file 4: Figure S3, both imprecise excisions disrupted the coding sequence and were expected to encode only a truncated protein containing the first ~158 N-terminal amino acids. Both excision derivatives had the same phenotypic effects as *GE24185* and were complemented by the *hsp83-dCTCF*^*+*^ transgene. These results support the conclusion that *GE24185* is a null allele of the *dCTCF* gene [55].

As was shown previously [55], adult flies homozygous for the *GE24185* mutation as well as the two excision derivatives had a mild but highly penetrant held out wing phenotype and thin bristles throughout the animal, and exhibited a series of homeotic phenotypes in posterior parasegments indicative of a loss of *Abd-B* activity. These homeotic phenotypes were temperature dependent. They were typically observed in flies raised at 25 °C, while they were much less frequent when the flies were raised at 18 °C. One of these phenotypes was the presence of a rudimentary A7 segment in males as would be expected for a loss-of-function transformation of PS12 into PS11 (Fig. 5d). Another was a protruding and rotated male genitalia. Also unlike wild-type males, *GE24185* males had bristles on the A6 sternite and sometimes also patchy pigmentation of the A5 tergite. The former phenotype is characteristic of a PS11 to PS10 transformation, while the latter is expected for a PS10 to PS9 transformation. While A7 and A8 do not form cuticular structures in adult males, they contribute to the cuticle in females. Homeotic transformations of the A7 sternite into A6 were evident in surviving *GE24185* females (Additional file 5: Figure S4). Adult mutant females had significantly reduced egg production, and produced no viable offspring when mated to homozygous mutant males. These lethal effects could, however, be rescued by mating the homozygous mutant females to heterozygous balancer males. This finding indicates that zygotic *dCTCF* expression can compensate for the absence of maternally derived dCTCF.

### Selective depletion of dCTCF from the bithorax complex in *GE241845* pupae

Mohan et al. (2007) found that though the levels of dCTCF were substantially reduced in *GE24185* larvae, maternally derived protein could still be detected at approximately 25 % of the sites in salivary gland polytene chromosomes that are normally observed in wild-type polytenes [55]. One idea suggested by this observation is that the homeotic transformations evident in *GE24185* adults arise because dCTCF is selectively lost from the bithorax complex (BX-C). When dCTCF depletion compromises BX-C insulator function, this might enable Polycomb response elements (PREs) in silenced *cis-*regulatory domains to repress neighboring active *cis-*regulatory domains and thus downregulate *Abd-B* expression in a manner that changes segmental identity. Alternatively, or in fact in addition, the proper functioning of the *Abd-B* promoter could require dCTCF.

**Fig. 6:**
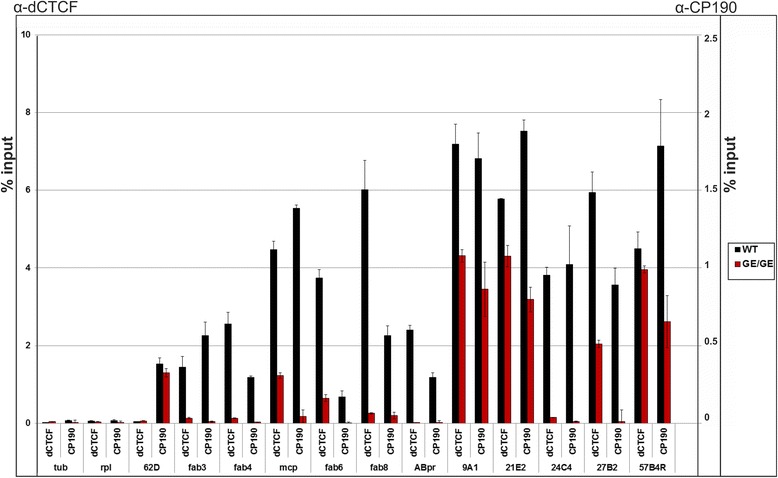
Histograms show dCTCF or CP190 occupancy in chromatin isolated from mid-late pupa at sequences containing the BX-C insulators *Fab-3*, *Fab-4*, *Mcp*, *Fab-6*, *Fab-8*, the *Abd-D* promoter, and several previously defined dCTCF insulators (9A1, 21E2, 24C4, 27B2 and 57B4R). Cross-linked chromatin prepared from wild-type (*WT*) (*y* ^*1*^ *w* ^*1118*^) pupae and homozygous *GE24185* (*GE/GE*) mutant pupae was immunoprecipitated with antibodies directed against the N-terminal domain of dCTCF and CP190. Sequences from tub, rpl32, and 62D regions were used as negative controls for dCTCF and CP190 association. 62D is an example of a sequence in which CP190 occupancy is independent of dCTCF. The left axis shows the scale for dCTCF enrichment, while the right axis shows the scale for CP190 enrichment. Each chromatin immunoprecipitation experiment was performed in at least two independent biological replicates. Error bars show standard deviations of quadruplicate PCR measurements. The results are presented as a percentage of input DNA

Mohan et al. [55] addressed this question by examining dCTCF association with BX-C in the brain of wild-type and *GE24185/Df(3L)0463* larvae and found that dCTCF was absent from most insulators in the complex, but was detected at *Abd-B* promoter. We have repeated these experiments using chromatin prepared from pupae because it is during this stage that the adult cuticle is elaborated. We selected six dCTCF binding sites from BX-C: the *Mcp*, *Fab-6*, *Fab-8* insulators [67–77], *Fab-3* region, *Fab-4* region, and the *Abd-B* promoter region (Fig. 6) [78]. We also selected the *CG1354* promoter region (9A1) [55] and four regions that were identified by Schwartz et al. [56] as requiring dCTCF to block the spread of H3K27me3 in the BGL3 cell line [79]. In addition to testing dCTCF association with these sequences, we also assayed CP190.

ChIP experiments with chromatin isolated from wild-type pupae using antibodies directed against the N-terminal region of dCTCF confirmed that it was bound to the insulators and the *Abd-B* promoter in BX-C and to the *CG1354* promoter (9A1) and four BGL3 insulators (Fig. 6). However, the extent of enrichment of *Fab-3*, *Fab-4*, and the *Abd-B* promoter was about half that of the other BX-C insulators (*Mcp*, *Fab-6*, and *Fab-8*) and also of the *CG1354* promoter (9A1) and four BGL3 insulators. As expected, the enrichment of the BX-C dCTCF sequences was substantially reduced in ChIPs of homozygous *GE24185* pupae. The extent of reduction was not, however, uniform. Near-background levels of dCTCF were observed for *Fab-3*, *Fab-4*, *Fab-8*, and the *Abd-B* promoter in *GE24185* mutant pupae, while residual dCTCF could still be detected at *Mcp* and, to a lesser extent, *Fab-6*. By contrast, only one BGL3 insulator, 24C4, showed a loss of dCTCF equivalent to that seen for most of the BX-C sequences. Three of the other insulators, 9A1, 21E2, and 57B4R, showed only very modest reductions. Though the loss of dCTCF at the fourth insulator, 27B2, was more substantial, the occupancy level in mutant pupae was still about the same as that seen for several of the BX-C elements in wild type.

The rather modest reductions in dCTCF evident at several non-BX-C insulators as well as the residual dCTCF that was retained at two of the BX-C insulators in the absence of a zygotic source of dCTCF protein would suggest that a significant amount of maternally derived dCTCF remains up to at least the pupal stage in *GE24185* mutant animals. Moreover, it would appear that the protein is preferentially retained at a subset of the *dCTCF* insulators. However, an alternative explanation for the apparent persistence of maternal dCTCF is that our antibody recognized some other protein species that happened to bind to the insulators that were pulled down in ChIPs of the mutant pupae. To exclude this possibility, we used an antibody directed against the C-terminal region of the dCTCF protein for ChIP experiments (Additional file 6: Figure S5). ChIPs with this antibody paralleled those obtained with the N-terminal antibody. Substantial amounts of dCTCF persisted at several non-BX-C insulators in *GE24185* mutant pupae, while there was still some residual dCTCF remaining at the BX-C insulators *Mcp* and *Fab-6*.

With the exception of *Mcp*, all of the BX-C insulators and the *Abd-B* promoter had less CP190 than the BGL3 insulators. The effects of *GE24185* mutation on CP190 association with the BX-C and BGL3 sequences also followed a pattern similar to that observed for dCTCF. For all of the BX-C insulators, loss of dCTCF was accompanied by a loss of CP190. For the other insulators, the reduction in CP190 occupancy was, with one exception, roughly comparable to that seen at the insulator for dCTCF. For example, dCTCF levels were reduced about 40 % for 9A1, while CP190 was reduced about 50 %. The one exception was 27B2, which lacked CP190 in *GE24185* mutant pupae, yet retained significant dCTCF occupancy. To confirm that the loss of CP190 occupancy at dCTCF insulators was not due to a reduction in CP190 protein levels, we probed western blots of extracts prepared from wild-type and *GE24185* mutant pupae (Additional file 7: Figure S6).

### Role of CTDs and NTDs in functional activity of the dCTCF protein

To examine the *in vivo* functions of the N-terminal multimerization domain and the C- terminal CP190 interacting domain, we generated *hsp83* transgenic lines expressing FLAGx3-tagged dCTCF proteins lacking these domains. For the multimerization domain, we deleted sequences between amino acid 90 and amino acid 170 (dCTCF^ΔN^). This deletion spans the region required for dCTCF–dCTCF interactions in vitro. For the CP190 interaction module, we used the C-terminal 774–818 deletion (dCTCF^ΔC^) that eliminates interactions between CTCF and CP190 in S2 cells (Fig. 5c). The activities of two independent transgenic lines expressing the deleted proteins were tested in the *GE24185* mutant background.

As described above, the control transgene, *dCTCF*^*+*^, encoding the wild-type protein fully complemented the zygotic and maternal effect lethality of the *GE24185* mutation (Fig. 5d). It also rescued the thin bristles phenotype and the loss-of-function homeotic transformations evident in PS11-14 (Fig. 5 and Additional file 5: Figure S4). However, in approximately 10 % of the *dCTCF*^*+*^ males we observed a partial loss of pigmentation in the tergite of abdominal segment A5, which is characteristic of a loss-of-function transformation of A5 (PS10) to A4 (PS9) transformation [80]. The held out wing phenotype was also not rescued. The *dCTCF*^*ΔC*^ transgenes resembled *dCTCF*^*+*^. They fully rescued the zygotic and maternal effect lethality of the *GE24185* mutations, the thin bristles, and the PS11-14 homeotic transformations in males and females. Like *dCTCF*^*+*^, we also observed a partial loss of pigmentation on the A5 tergite; however, the frequency was somewhat higher (50 % as compared to 10 %) and the size of the depigmented patches was typically larger. In contrast to *dCTCF*^*ΔC*^, the *dCTCF*^*ΔN*^ transgene only partially ameliorated the zygotic and maternal effect lethality of *GE24185* and *dCTCF*^*ΔN*^ transgenic flies had reduced viability and were only semi-fertile. In addition, *dCTCF*^*ΔN*^ did not rescue the thin bristles phenotype or the homeotic transformation seen in the abdominal segments of *GE23185* adult males and females (Fig. 5d and Additional file 5: Figure S4).

### Chromatin association of dCTCF^+^, dCTCF^ΔC^, and dCTCF^ΔN^

As a prelude to analyzing the chromosome association of the mutant dCTCF proteins, we first examined the expression of transgenic wild-type and mutant dCTCF proteins. For this purpose we probed fly extracts with antibodies directed against the FLAG tag. As shown in the western blot in Additional file 8: Figure S7, the mutant proteins were expressed at nearly equivalent levels. When we probed western blots with antibodies directed against dCTCF, we found that the levels of proteins produced by the transgenes were about twofold less than that of the endogenous gene (not shown). This would suggest that the incomplete rescue of two of the GE24185 phenotypes (loss of A5 pigmentation and held out wings) by the *hsp83:dCTCF*^*+*^ transgene is likely due, at least in part, to the insufficient expression of dCTCF.

**Fig. 7:**
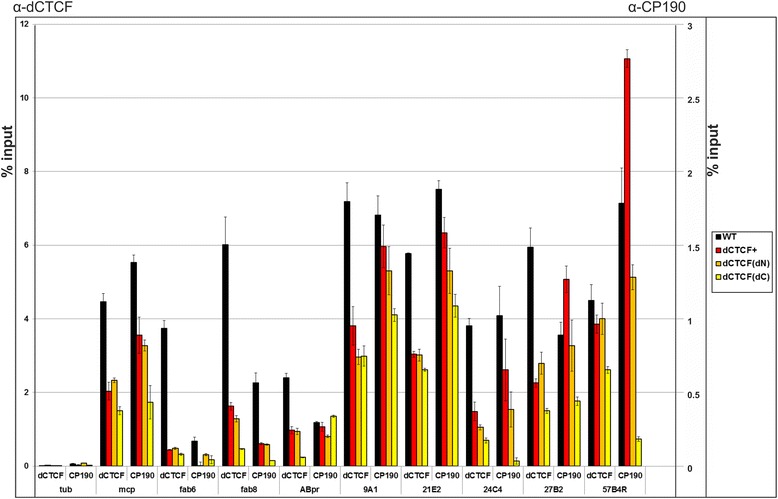
Histograms show dCTCF or CP190 occupancy in chromatin from mid-late pupa at sequences containing the BX-C insulators *Mcp*, *Fab-6*, *Fab-8*, the *Abd-B* promoter, and several other previously defined dCTCF insulators (9A1, 21E2, 24C4, 27B2, 57B4R). Chromatin was isolated from homozygous *GE24185* mutant pupae that also carry the *hsp83:dCTCF* ^*+*^, *hsp83:dCTCF* ^*ΔN*^ *,* or *hsp83:dCTCF* ^*ΔC*^ transgenes. The tub sequence was used as the negative control. The left axis shows the scale for dCTCF enrichment, while the right axis shows the scale for CP190 enrichment. Each ChIP experiment with 2- to 3-day pupae was performed in at least two independent biological replicas. Error bars show standard deviations of quadruplicate PCR measurements. The results are presented as a percentage of input DNA. *WT* wild type

Next, we examined the association of transgenic wild-type and deletion mutant dCTCF with the insulators and *Abd-B* promoter in BX-C, *CG1354* promoter (9A1), and the BGL3 insulators. In ChIPs of chromatin isolated from *GE24185 hsp83*:*dCTCF*^*+*^ pupae we found that the occupancy levels of the transgenic dCTCF^+^ at most of these sites were reduced about twofold compared to the endogenous protein in wild-type flies (Fig. 7). This reduction would be consistent with the lower levels of dCTCF in the *GE24185 hsp83:dCTCF*^*+*^ flies. However, there were three exceptions. For two of these, the *Fab-6* and *Fab-8* insulators, the reductions in dCTCF occupancy were greater than twofold. dCTCF occupancy at *Fab-6* was reduced by nearly tenfold while it was reduced by almost fourfold at *Fab-8.* While *Fab-8* still retained levels of dCTCF comparable to several other BX-C sites, only a small amount of dCTCF was detected at *Fab-6*. Because one function of the *Fab-6* insulator in PS10 cells is to prevent inactivation of the *iab-5 cis-*regulatory domain by blocking the spread of Polycomb-dependent silencing from the PRE in the adjacent *iab-6 cis-*regulatory domain, the substantial reduction in dCTCF association with the *Fab-6* insulator could potentially account for the persistence of the A5–A4 transformation in a subset of the *GE24185* flies rescued by *hsp83:dCTCF*^*+*^. The other exception, the BGL 57B4R insulator, had near wild-type levels of dCTCF.

Like *dCTCF*^*+*^, *dCTCF*^*ΔN*^ and *dCTCF*^*ΔC*^ occupancy at dCTCF sites in BX-C and the BGL3 insulators was reduced compared to wild type (Fig. 7). At most sites, the levels of *dCTCF*^*ΔN*^ occupancy were very close to those for *dCTCF*^*+*^. In contrast, *dCTCF*^*ΔC*^ occupancy levels were in most instances slightly lower (less than twofold) than either *dCTCF*^*+*^ or *dCTCF*^*ΔN*^. Though the effects were small, they suggest that chromatin association of the dCTCF C-terminal deletion was partially compromised. Because this domain mediates interactions with CP190, this finding would support the idea that dCTCF binding to chromatin can be stabilized by interactions with CP190.

### CP190 occupancy requires dCTCF but not necessarily the dCTCF-CTD

We also tested whether the reductions in CP190 occupancy evident in *GE24185* mutants could be rescued by the *hsp83:dCTCF* transgenes encoding the wild-type and mutant proteins. Supporting the idea that dCTCF functions in CP190 recruitment, we found that *dCTCF*^*+*^ and *dCTCF*^*ΔN*^ promote CP190 occupancy at sites bound by dCTCF in vivo (Fig. 7). For BX-C insulators the effects on CP190 occupancy seemed to correlate with the levels of the transgene dCTCF associated with the insulator. For example, at *Mcp* where the *dCTCF*^*+*^ and *dCTCF*^*ΔN*^ transgene proteins were present at only about half the level of the endogenous dCTCF, CP190 occupancy was about 60 % of wild type (see Fig. 7). Similarly at *Fab-8*, the transgene dCTCF proteins and CP190 were present at levels about 30 % that of wild type. CP190 occupancy for two BGL3 insulators, 24C4 and 27B2, also depended upon dCTCF. In *GE24185* mutants, CP190 was not detected at either of these insulators, while association was restored by the *dCTCF*^*+*^ and, to a somewhat lesser extent, the *dCTCF*^*ΔN*^ transgenes. Because the three other insulators (9A1, 21E2, and 57B1R) retained significant levels of both dCTCF and CP190 in *GE24185* mutants, it was not clear whether dCTCF is essential for CP190 occupancy or if it is one of several factors that contribute to CP190-insulator association. Thus, though CP190 occupancy in *dCTCF*^*+*^ and *dCTCF*^*ΔN*^ flies at these three insulators was near wild type, the extent to which transgene dCTCF protein contributed to the rescue was not entirely clear.

Further insight into the role of dCTCF in CP190 occupancy came from ChIPs of *dCTCF*^*ΔC*^ transgene embryos (Fig. 7). There seemed to be three classes with respect to the requirement for the dCTCF-CTD. In the first class were the BGL3 insulators 24C4 and 54B4R and also *Fab-8*. In this class, CP190 occupancy required the dCTCF-CTD and was substantially reduced in *dCTCF*^*ΔC*^ transgene flies compared to wild type or the two other *dCTCF* rescue transgenes (Fig. 7). The second class was represented by *Mcp* and 27B2. Like 24C4, 54B4R and *Fab-8*, CP190 occupancy at these two insulators depended upon dCTCF and was reduced to near-background levels in *GE24185* flies. However, unlike the insulators in the first class, the *dCTCF*^*ΔC*^ transgene could partially rescue CP190 association. In the third class was the *Abd-B* promoter. Although CP190 occupancy at the *Abd-B* promoter required dCTCF (see Fig. 6), the requirement seemed to be independent of the dCTCF-CTD and was fully rescued by the *dCTCF*^*ΔC*^ transgene.

### dCTCF is required to properly initiate Abd-B expression in the embryo

The visible phenotypic defects in *GE24185* adult flies arise from alterations in the patterns of gene expression induced by the gradual depletion of maternal dCTCF as the animals develop. It seemed possible that the effects on gene regulation might differ if dCTCF were completely absent at the onset of embryonic development instead of being present at near-normal levels and then slowly lost. To explore this possibility, we examined the expression of three genes, the homeotic gene *Abd-B*, the segment polarity gene, *engrailed* (*en*) [81], and the *Notch* pathway gene, *insensitive* (*insv*) [82] in the progeny of *GE24185* mothers and fathers. Unlike the progeny of heterozygous parents, these embryos lack both maternal and zygotic dCTCF. Because the greatly reduced fecundity of *GE24185* mothers made embryo collections problematic, we restricted our analysis to mid-embryogenesis.

**Fig. 8:**
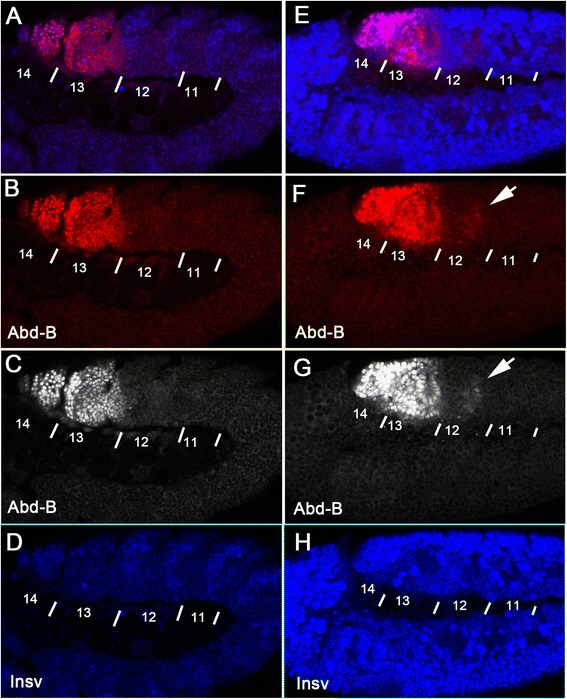
Expression of Abd-B and Insv in stage 10/11 wild-type and *dCTCF* ^*m-z-*^ embryos. Stage 11 wild-type and stage 10 *dCTCF* ^*m-z-*^ (from cross of homozygous *GE24185* parents) embryos were probed with antibodies directed against Abd-B (mouse monoclonal 1A2E9 from Developmental Studies Hybridoma Bank) and Insv (a rabbit polyclonal: gift of Tsutomu Aoki) and visualized by confocal microscopy. Parasegments are indicated in the Fig. *Arrows* in panels F and G indicate Abd-B expression in PS12. **a** Wild type: merged image. **b** Wild type: Abd-B. **c** Wild type: Abd-B. **d** WT: Insv. **e** *dCTCF* ^*m-z-*^: merged image. **f** *dCTCF* ^*m-z-*^:Abd-B. **g** *dCTCF* ^*m-z-*^:Abd-B. **h** *dCTCF* ^*m-z-*^: Insv. *Red/Gray* Abd-B, *Blue* Insv

**Fig. 9:**
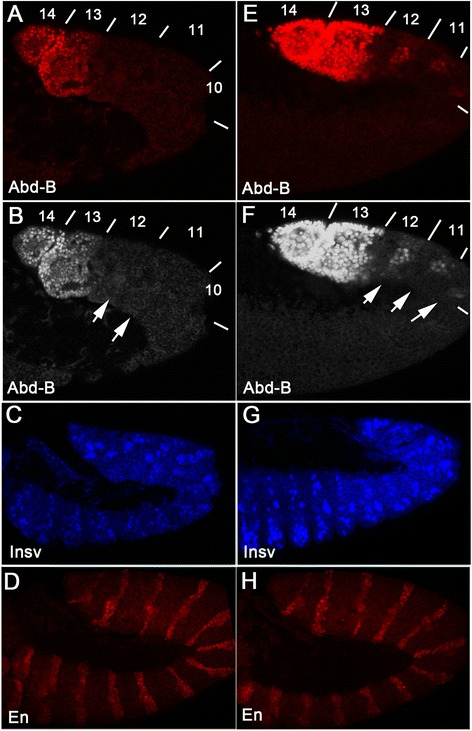
Expression of Abd-B, Insv, and En in stage 12 wild-type and *dCTCF* ^*m-z-*^ embryos. Stage 12 wild type (**a-d**) and *dCTCF* ^*m-z-*^ (**e-h**) were probed with antibodies directed against Abd-B (panels a,b e, and f), Insv (panels c and g), and En (panels d and h). *Arrows* in panel f point to Abd-B protein expression in PS12, PS11, and PS10 in the stage 12 *dCTCF* ^*m-z-*^ embryo. By contrast, *arrows* in panel b indicate that little or no Abd-B was detected in PS12 or PS11 of the wild-type embryo. See text for details

The pattern of Abd-B expression during mid-embryogenesis in wild-type embryos is dynamic [83, 84]. In stage 10 germ band extended embryos, Abd-B protein is expressed in parasegments PS13 and PS14, while little or no protein is evident in more anterior parasegments. Abd-B protein first begins to accumulate at detectable levels in more anterior parasegments towards the end of stage 11 at the onset of germ band retraction. Only a low level of protein is initially observed in PS12. As the germ band retracts, Abd-B levels increase in PS12, and protein begins to accumulate at detectable levels in PS11. Finally at the end of germ band retraction in stage 13, low levels of Abd-B are found in PS10. Panels E-G in Fig. 8 show the pattern of Abd-B expression in a stage 10 *dCTCF*^*m-z-*^ embryo. For the purposes of comparison, a slightly older stage 11 wild-type embryo is shown in panels A-C. Abd-B expression in the *dCTCF*^*m-z-*^ embryo differed in two respects from wild type. First, the levels of Abd-B in both PS13 and PS14 of the *dCTCF*^*m-z-*^ embryo were noticeably higher than that found in the corresponding parasegments of the wild-type embryo (compare panels C and G). Second, while Abd-B could not be detected in PS12 in the stage 11 wild-type embryo, it was prematurely expressed in PS12 in the stage 10 *dCTCF*^*m-z-*^ mutant embryo. The differences in both timing and level of expression seen in stage 10/11 wild-type and *dCTCF*^*m-z-*^ embryos were also evident in older embryos. In the stage 12 wild-type embryo shown in Fig. 9a,b, there was at most only a very low level of Abd-B protein in PS12, while Abd-B did not appear to be expressed in PS11. In contrast, Abd-B was readily detected in both PS12 and PS11 of the *dCTCF*^*m-z-*^ embryo. Moreover, protein could even be seen in a cluster of cells in PS10. In addition to being prematurely expressed in more anterior parasegments, the level of Abd-B protein in PS13 and PS14 was higher than that in the wild-type control [83, 84].

The segment polarity gene *en* and the Notch pathway gene *insv* were expressed in a stripe-like pattern in each parasegment in the ectoderm of wild-type germ band extended embryos; however, while all of the cells in the *en* stripes appeared to express essentially the same levels of En protein (Fig. 9d,h) only a subset of the cells in the *insv* stripes expressed Insv (Figs. 8d and 9c). In the case of En, there were no obvious changes in the stripe pattern or in the level of protein in the cells expressing En in the *dCTCF*^*m-z-*^ mutant. In contrast, there was a substantial increase in the number of cells that expressed Insv in *dCTCF*^*m-z-*^ embryos. The level of Insv protein in these cells also appeared to be elevated. These changes were evident in both the stage 10 embryo in Fig. 8h and the stage 12 embryo in Fig. 9h.

We also examined the expression of Abd-B in *GE24185* embryos rescued by the *hsp83:dCTCF*^*+*^, *hsp83:dCTCF*^*ΔN*^, and *hsp83:dCTCF*^*ΔC*^ transgenes. The pattern of Abd-B expression in *GE24185 hsp83:dCTCF*^*+*^ transgenic embryos resembled wild type. This was also true for *GE24185 dCTCF*^*ΔC*^ transgenic embryos. In the case of *hsp83:dCTCF*^*ΔN*^, we occasionally observed stage 11–15 embryos that appeared to have slightly elevated levels of Abd-B.

## Discussion

CTCF is one of only a few insulator/chromosomal architectural DNA binding proteins that are known to be common to both insects and vertebrates [28, 30, 36, 85]. While it has been implicated in a wide range of nuclear functions ranging from gene expression to recombination and replication, a thread that seems common to most of its known activities is one of organizing the chromatin fiber. With the aim of better understanding its biological activities, we have begun a systematic analysis of the *Drosophila melanogaster* CTCF protein, dCTCF. In the studies reported here we have focused on two modules in the protein, one that mediates multimerization of dCTCF and the other that mediates interactions with the chromosomal protein CP190.

Using a combination of biochemical procedures we localized the dCTCF multimerization module to the N-terminus of the protein, spanning a region of about 100 amino acids between 70 and 163. At least part of this region (84–188) appears to be highly structured in solution as it is protease resistant. Although further studies will be required, our data would be most consistent with a model in which the multimerization module mediates the formation of tetrameric rather than dimeric dCTCF complexes. However, in either case, multimerization should enable dCTCF to interact simultaneously with several closely spaced recognition motifs because is found in insulators like *Fab-3*, *Fab-6*, and *Fab-8*. Presumably this could stabilize dCTCF association with chromatin by increasing the half-life of the protein–DNA complex. Alternatively, or in addition, the dCTCF multimers could bind simultaneously to dCTCF sites in different insulators, generating a chromatin loop linked together at its base by the insulator bound dCTCF multimeric complexes. A direct linkage of distant dCTCF binding sites by the dCTCF multimers would be consistent with the insulator bypass experiments using pairs of the appropriately oriented multimerized dCTCF binding sites [15]. Like its insect counterpart, the vertebrate CTCF protein has also been implicated in the formation of chromatin loops [28]. Because vertebrate CTCF is capable of self-association [58], it is possible that distant CTCF binding sites are linked together via some sort of CTCF multimeric complex. However, unlike the NTD of the *Drosophila* protein, biochemical studies suggest that the NTD of vertebrate CTCF is monomeric and disordered [86]. Thus, multimer formation would have to involve other domains. In this regard it is of interest that Pant et al. [59] found pairwise interactions between the C-terminal zinc finger domains of the vertebrate CTCF protein.

We also analyzed how dCTCF and CP190 interact with each other. Yeast two-hybrid experiments and GST-pull-downs map the CP190 interaction module of dCTCF to the CTD. For CP190, the primary dCTCF interaction module was associated with the CP190 BTB domain. Previous studies have shown that the CP190 BTB domain forms a highly stable dimer [51, 61]. If there is a one-to-one relationship between the dCTCF-CTD–CP190 interaction module and the CP190 BTB domain, this CP190 BTB dimer could link two dCTCF multimers together, potentially building higher order protein–DNA complexes. However, our experiments do not favor a one-to-one interaction between dCTCF and CP190. Instead, our results would support a model in which a single dCTCF-CTD–CP190 interaction module associates preferentially with a dimerized CP190 BTB domain to give a 2xBTB^CP190^:dCTCF-CTD heterotrimer. This appears to be true, at least in these in vitro experiments, even when dCTCF is present in excess. Though confirmation will require a direct structural analysis of the dCTCF–CP190 complex, these findings would argue that the BTB dimer of CP190, by itself, could not function as the bridge linking two distant dCTCF insulators together. However, CP190 has additional domains (aspartic acid-rich D and microtubule targeting domains) that are involved in interaction with the DNA binding proteins like the insulator protein ZIPIC [60, 87]. Thus, CP190 might form complexes with or bridges between different insulator proteins like ZIPIC and dCTCF that might stabilize binding to chromatin. Because CP190 has a sequence non-specific zinc finger DNA BD [52], an alternative function for CP190 might be to stabilize dCTCF association with chromatin by binding (non-specifically) to sequences adjacent to dCTCF binding sites. However, because there are only very modest effects at most on dCTCF occupancy when the C-terminal dCTCF CP190 interaction domain is deleted, such a function would not seem to be critical.

To complement these biochemical studies, we examined the functioning of the dCTCF multimerization and CP190 interaction modules in vivo. Previous studies have shown that maternally derived dCTCF is sufficient to sustain development through to the adult stage [55]. However, the viability of homozygous *GE24185* animals, which lack zygotic *dCTCF*, was significantly reduced and the surviving adult flies exhibited several characteristic phenotypes. These included thin bristles, held out wings, abdominal segmentation defects, a substantially reduced egg production, and a maternal effect lethality. We found that an *hsp83* transgene expressing the wild-type protein rescued all but two of the phenotypic effects of the *GE24185* mutation. The two exceptions were the held out wing phenotype and the presence, in a small fraction of the *hsp83:dCTCF*^*WT*^ adults, of a weak A5 (PS10) to A4 (PS9) transformation. It seems likely these two remaining phenotypes were due to the fact that the level of dCTCF expressed by the transgene is less than that produced by the endogenous gene. In the case of the A5 to A4 transformation, this suggestion fits with both the roughly tenfold reduction in dCTCF occupancy at the *Fab-6* insulator and the known role of this insulator in *Abd-B* regulation. *Fab-6* functions to protect the *Abd-B cis-*regulatory domain specifying PS10 (A5), *iab-5*, from the adjacent PS11 *cis-*regulatory domain, *iab-6* [69, 77, 88, 89]. When only reduced levels of dCTCF are present, it is possible that Polycomb group (PcG) complexes may spread into *iab-5* from *iab-6* silencing the *iab-5 cis-*regulatory domain inappropriately in PS10.

Somewhat surprisingly, a dCTCF protein, dCTCF^ΔC^, lacking sequences in the CTD critical for interactions with CP190 was just about as effective as the wild-type protein in complementing the *GE24185* mutant. Like wild type, it rescued the zygotic and maternal effect lethality, the egg production defect, and the bristle phenotypes, but not the held out wing or the A5 to A4 transformation. ChIP experiments were consistent with the idea that CP190 interactions may help stabilize dCTCF association with chromatin because dCTCF occupancy levels were reduced in *hsp83:dCTCF*^*ΔC*^ pupae (compared to *hsp83:dCTCF*^*WT*^) at most of the insulators we examined. While the effects of the mutation on dCTCF occupancy were modest, much greater reductions in CP190 levels were evident at least at some sites (e.g., the BGL3 insulators, 24C and 57B4R; Fig. 6). At these sites it would appear that CP190 occupancy required the dCTCF C-terminal CP190 interaction module. However, this was not always the case. For example, at *Mcp*, the presence of the mutant dCTCF^ΔC^ protein was sufficient to ensure CP190 occupancy (Fig. 6). Because CP190 occupancy at *Mcp* required dCTCF (see Fig. 5), it would appear that dCTCF can promote CP190 association by a mechanism that is independent of its C-terminal CP190 interaction module. Recently, Pita was found to bind *Mcp* and recruit CP190 [87]. The binding of the two proteins to *Mcp* are interdependent. One obvious possibility is that dCTCF^ΔC^ binding to *Mcp* stabilizes the association of Pita, which in turn can recruit CP190.

While the dCTCF^ΔC^ protein was just about as effective as wild-type dCTCF in rescuing the *GE24185*, this was not true for dCTCF^ΔN^. This mutant protein only partially ameliorated the zygotic and maternal effect lethality, and surviving adult flies exhibited the same visible phenotypes as the *GE24185* mutant. Because occupancy levels of the dCTCF^ΔN^ protein were nearly equivalent to that of dCTCF^WT^, it would appear that some other activity of the dCTCF protein must be partially compromised by the deletion of the multimerization domain. One possibility would be a function in linking distant dCTCF insulators together; however, this region of the protein could have other activities besides mediating dCTCF dimerization. Importantly, because CP190 occupancy at BX-C insulators and the *Abd-B* promoter was similar to that in *hsp83:dCTCF*^*WT*^, *Abd-B-*dependent abdominal phenotypes in adult *hsp83:dCTCF*^*ΔN*^ flies (and, by inference, *GE24185* flies) may arise by a mechanism that is independent of CP190.

Because there is a substantial maternal contribution of dCTCF, the abdominal segmentation defects in surviving *GE24185* flies were likely a consequence of a gradual reduction in dCTCF occupancy at the *Abd-B* insulators and/or promoter as the animals developed. Significantly, eliminating dCTCF at the onset of embryonic development had a quite different effect on *Abd-B* gene activity. Instead of being reduced, *Abd-B* expression was substantially upregulated in the posterior parasegments of *dCTCF*^*m-z-*^ embryos. These paradoxical effects on *Abd-B* regulation would not be consistent with a primary function for dCTCF in the intrinsic activity of the *Abd-B* promoter—the adult phenotype would require dCTCF to function as an activator, while the embryonic phenotype would require dCTCF to function as a repressor. Rather, one would imagine that whatever role dCTCF plays at the *Abd-B* promoter (positive or negative), this function is likely to be the same throughout development. For this reason, the opposing stage-specific effects on *Abd-B* activity are more readily explained by the quite different modes of regulation of the *Abd-B* (*iab*) *cis-*regulatory domains in embryos and in larvae/pupae [88, 89].

In larvae and pupae, *Abd-B* regulation is in the maintenance phase and depends upon the PREs in each *iab cis*-regulatory domain [73, 88–91]. The PREs function to recruit PcG proteins, keeping inactive *cis-*regulatory domains *off* in the parasegments where they should be silenced. For this reason, a gradual reduction in insulator activity in the larval and pupal stages could result in the spreading of PcG silencing from PREs in inactive *cis-*regulatory domains to their flanking active neighbors and the consequent downregulation of *Abd-B* gene activity in posterior parasegments. (Of course, if *dCTCF* were also required for *Abd-B* promoter activity, this would tend to enhance any loss-of-function phenotypes associated with the spread of silencing in the *cis-*regulatory domains in *dCTCF*^*z-*^ mutants. Conversely, these loss-of-function phenotypes would be suppressed if *dCTCF* represses instead of enhances *Abd-B* promoter activity). By contrast, in *dCTCF*^*m-z-*^ embryos*,* dCTCF would be absent when the gap and pair-rule genes initially establish the parasegment-specific patterns of *Abd-B* gene activity during the blastoderm–early gastrula stage [92–95]. At this point in development one of the key functions of insulators is to prevent cross talk between the adjacent parasegment-specific initiation elements that can inappropriately activate or silence the *cis*-regulatory domains flanking the insulator [67, 69, 77, 96]. For mutations that disrupt specific insulators, this mix of ectopic activation and silencing can be visualized in the embryo by changes in the level of *Abd-B* expression [67, 77, 96]. For example, in *Fab-6* mutants, the inappropriate activation of the *iab-6 cis-*regulatory domain in PS10 would noticeably upregulate *Abd-B* expression in this parasegment compared to that in wild type where *iab-5* normally directs *Abd-B* expression [69]. However, the level of *Abd-B* expression in PS10 in the *Fab-6* mutant is not equivalent to that in the adjacent parasegment PS11 because in some of the PS10 cells the *iab-5 cis-*regulatory domain is inappropriately silenced. In fact, this is the phenotype that we observed in *dCTCF*^*m-z-*^ mutant embryos. *Abd-B* expression is each posterior parasegment was upregulated compared to the corresponding parasegment in wild type, but the extent of upregulation was not equivalent to that in the adjacent more posterior parasegments.

While the opposing phenotypes associated with the loss *dCTCF* activity at different stages of development argue in favor of the idea that dCTCF is critical to the functioning of the insulators associated with the *Abd-B cis*-regulatory domains, there was one rather puzzling observation—namely, the apparent effects of the *dCTCF GE24185* mutation on the activity of the *Fab-7* insulator. In adult *GE24185* flies, the partial transformation of A6 (PS11) into A5 (PS10) would point either to a loss of *Abd-B* promoter activity or the spreading of silencing from *iab-7* PRE into *iab-6*, shutting off this *cis*-regulatory domain in PS10 (or a combination of both). In the embryo, the upregulation of *Abd-B* expression in PS11 would require either an increase in *Abd-B* promoter activity or the ectopic activation of *iab-7* by the *iab-6* initiator in PS11 cells. While these stage-specific phenotypic effects are more readily explained by a disruption in *Fab-7* insulator activity rather than diametrically opposite effects on the *Abd-B* promoter as development proceeds, *Fab-7* differs from the other *Abd-B* insulators in that it does not have dCTCF binding sites [78]. In this case, the apparent loss of *Fab-7* insulator activity would have to be an indirect consequence of disruptions in the functioning of the neighboring dCTCF-dependent insulators, *Fab-6* and *Fab-8*. Although transgene assays have argued that the activity of an insulator depends upon its neighbors (for review see [1]), this would be one of the first examples in an endogenous setting. Further studies will clearly be required to explain the apparent effects on *Fab-7* activity.

Additionally, we found that the expression of the *Notch* pathway gene, *insv*, was also changed in *dCTCF*^*m-z-*^ embryos, while *en* expression seemed to be unaffected. Unlike *Abd-B*, where there are good reasons to believe that the effects on gene regulation are a direct consequence of the loss of *dCTCF* activity, we do not know whether the effects on *insv* are direct or indirect. However, it is worth noting that the *insv* gene and its partner, *elba2*, are flanked by sequences that are bound by dCTCF, while the potential insulators for the *en* locus appear to be occupied by Su(Hw) rather than dCTCF [56, 57].

## Conclusions

CTCF is one of the few DNA binding insulator proteins that is conserved in bilaterians. To learn more about its role in chromosome architecture we have identified and characterized the two protein–protein interaction modules in *Drosophila* dCTCF. The first is responsible for the multimerization of the dCTCF protein while the second is responsible for interactions between dCTCF and CP190. We have also tested the functioning of proteins lacking these interaction modules in vivo. We found that a dCTCF protein lacking sequences critical for CP190 interactions was almost as effective as wild type in rescuing the phenotypic effects of a *dCTCF* null allele. In contrast, a dCTCF protein lacking the multimerization domain had only partial functionality and did not fully rescue phenotypic effects of the null allele.

## Methods

### Plasmid construction

For protein purification purposes, protein fragments were either PCR-amplified using corresponding primers (see Additional file 9: Table S2), or digested from dCTCF cDNA (1–288, *Bam*HI-*Eco*RI; 1–205, *Bam*HI-*Xho*I; 1–163, *Bam*HI-*Pvu*II; 1–125, *Bam*HI-*Rsa*I; 70–163, *Mbo*I-*Pvu*II; 125–180, *Rsa*I-*Rsa*I) and subcloned into pGEX-4T1 (GE Healthcare, Little Chalfont, Buckinghamshire, United Kingdom) or pET32a(+) vector (Merck Biosciences, Darmstadt, Germany) in-frame with corresponding tag. In the case of 6xHis-fusions without thioredoxin, its coding sequence was excised from pET32a(+) vector with *Nde*I. A modified pET32a(+) vector was used to express proteins with a TEV-cleavable thioredoxin-6xHis-tag.

For protein expression in S2 cells and for generation of transgenic flies, protein coding sequences were cloned in-frame with 3xFLAG, excised, and subcloned into the Casper vector with Hsp83 promoter [64].

### Generation and analysis of transgenic lines

The construct and P25.7wc plasmid were injected into *yacw*^*1118*^ pre-blastoderm embryos [97]. The resultant flies were crossed with *yacw*^*1118*^ flies, and the transgenic progeny were identified by their eye color.

### Protein expression and purification, size-exclusion chromatography, and chemical cross-linking

Protein expression and purification were performed using standard procedures. Briefly, BL21 cells were disrupted by sonication in buffer A (40 mM HEPES-KOH pH 7.7, 400 mM NaCl, 5 mM β-mercaptoethanol, 20 mM imidazole) containing 1 mM PMSF and Calbiochem Complete Protease Inhibitor Cocktail VII (1 μL/1 ml). After centrifugation, lysate was applied to an Ni-NTA column, and, after washing, was eluted with 300 mM imidazole and dialyzed against an appropriate buffer. For cleavage of the thioredoxin-6xHis-tagged protein, TEV protease was added at a molar ratio of 1:50 directly to the eluted protein. The mixture was incubated for 2 h at room temperature, dialyzed against buffer A, and applied to the Ni-NTA column. Flow-through was collected; dialyzed against 20 mM Tris–HCl pH 7.4, and 1 mM DTT; and further purified using a SOURCE15Q 4.6/100 column (GE Healthcare). Size-exclusion chromatography was performed as described [61] using Sephacryl S200 16/60 or Superdex 200 10/300GL columns (GE Healthcare) in 20 mM Tris–HCl pH 7.4, 150 mM NaCl, and 1 mM DTT. The protein concentration was adjusted to 5 μM and chemical cross-linking was carried out for 10 min at room temperature in buffer B containing 20 mM HEPES-KOH pH 7.7, 150 mM NaCl, 20 mM imidazole, and 1 mM β-mercaptoethanol. Cross-linking was quenched with 50 mM glycine and the cross-linked samples were resolved using SDS-PAGE followed by silver-staining. For analysis of complex formation between CP190-BTB and CTCF-CTD, the proteins were purified, dialyzed against buffer B, and mixed in corresponding molar ratio, with a constant CP190-BTB concentration of 20 μM. After incubation at room temperature for 2 h the protein mixture was cross-linked with glutaraldehyde as described above. Samples were resolved using SDS-PAGE followed by Coomassie staining.

### Pull-down assays

GST-pull-downs were performed with Immobilized Glutathione Agarose (Pierce) in buffer C (20 mM HEPES-KOH pH 7.7, 150 mM NaCl, 10 mM MgCl_2_, 0.1 mM ZnCl_2_, 0.1 % NP40, 10 % (w/w) glycerol). BL21 cells were grown in lysogeny broth media to an A600 of 1.0 at 37 °C and then induced with 1 mM IPTG at 18 °C overnight. ZnCl_2_ was added to a final concentration of 100 μM before induction. Cells were disrupted by sonication, centrifuged, and applied to resin for 10 min at room temperature. After binding, the resin was washed two times with buffer C. The resin with immobilized protein was then mixed with a solution of interacting protein or Schneider 2 cells nuclear lysate equilibrated in buffer C. Incubation was continued for 2 h at room temperature (for recombinant proteins) or at +4 °C (Schneider 2 cells nuclear lysate). The resin was then washed four times with buffer C containing 500 mM NaCl and elution performed with 50 mM reduced glutathione and 100 mM Tris pH 8.0, for 15 min.

### Limited proteolysis

Thioredoxin-fused CTCF[1–205] protein was purified and dialyzed against 20 mM HEPES-KOH pH 7.7, 150 mM NaCl, 10 mM MgCl_2_, and 2.5 mM CaCl_2_. The protein concentration was adjusted to 10 μM and indicated amounts of proteinase K (Fermentas) or trypsin (Sigma) diluted in the same buffer were added. After 10 min incubation at room temperature, PMSF was added to final concentration of 5 mM and incubation continued for a further 10 min. Samples were resolved using SDS-PAGE followed by Coomassie staining. The protein bands were excised and subjected to complete trypsin digestion and MALDI-TOF mass spectrometry. Protein identification and peptide mapping were performed using the MASCOT server (Matrix Science).

### *Drosophila* cells nuclear lysate preparation

Schneider 2 cells were grown in SFX media, collected by centrifugation at 700 g for 5 min, washed once with 1xPBS, resuspended in buffer IP-0 (10 mМ Tris–HCl pH 8.0, 10 mМ NaCl, 10 mМ MgCl_2_, 1 mМ EDTA, 1 mМ EGTA, 1 mМ DTT, 250 mМ sucrose, 1 mМ PMSF, 0.2 % NP-40, Calbiochem Protease Inhibitor Cocktail V), incubated for 10 minutes at +4 °C, and disrupted by 20 strokes in Dounce homogenizer on ice. Nuclei were collected at 3000 g for 10 min and resuspended in IP-10+ (10 mМ Tris–HCl pH 8.0, 10 mМ NaCl, 10 mМ MgCl_2_, 1 mМ EDTA, 1 mМ EGTA, 1 mМ DTT, 10 % glycerol, 0.1 % NP-40; in the case of subsequent immunoprecipitation assays, DTT was not used). An equal volume of IP-850+ (10 mМ Tris–HCl pH 8.0, 850 mМ NaCl, 10 mМ MgCl_2_, 1 mМ EDTA, 1 mМ EGTA, 1 mМ DTT, 10 % glycerol, 0.1 % NP-40) was added and nuclei were lysed for 10 min on ice, after that two volumes of IP-10+ and 1 U/ml DNAse I were added and incubation was continued for 10 min in a rotator at room temperature. After centrifugation at 16,000 g for 20 min, lysate was used in immunoprecipitation or GST-pull-down assays.

### Co-immunoprecipitation

Antibodies were immobilized for 2 h on pre-equilibrated Protein A or Protein G beads in buffer D (20 mM Tris pH 7.4, 150 mM NaCl, 10 mM MgCl_2_, 0.1 mM ZnCl_2_, 0.1 % NP40, 10 % (w/w) glycerol). The beads were then washed for 1 h with the same buffer containing 10 mg/ml BSA, and incubated overnight at +4 °C with nuclear lysate. After incubation with the lysate, the beads were washed four times with buffer D containing 500 mM NaCl and boiled in SDS-PAGE sample buffer.

### Fly extract preparation

Twenty adult flies were homogenized with a pestle in 200 μl of 1xPBS containing 1 % β-mercaptoethanol, 10 mM PMSF, and 1:100 Calbiochem Complete Protease Inhibitor Cocktail VII. The suspension was sonicated three times for 5 s at 5 W. Then, 200 μl of 4xSDS-PAGE sample buffer was added and the mixture incubated for 10 min at 100 °C and centrifuged at 16,000 g for 10 min.

### Antibodies

Antibodies were raised against dCTCF[1–163], dCTCF[612–818], and CP190[308–1096] fragments in rabbits and rats. GAGA-factor antibodies were raised against full-length protein in rats. Antibodies were purified from serum by ammonium sulfate fractionation followed by affinity purification using CNBr-activated Sepharose (GE Healthcare) with standard protocols. Other antibodies were anti-FLAG (M2, Sigma), anti-6xHis (GE Healthcare), anti-Abd-B (Iowa Developmental Studies Hybridoma Bank), and anti-Insv (gift of Tsutomu Aoki).

### Cell culture, transfection, and dual luciferase assay

*Drosophila* S2 cells were grown in SFX medium (HyClone) at 25 °C. Transfection of plasmids was performed with the Cellfectin II reagent (Invitrogen) according to the manufacturer’s instructions. Typically, cells were transfected in six-well plates and grown for 24–48 h before harvesting. All transfection procedures were performed with three independent replicates.

The dual luciferase assay was performed with the Firefly & Renilla Luciferase Assay Kit (Biotium).

### Chromatin immunoprecipitation

Chromatin was prepared from S2 cells and mid-late pupae.

#### S2 cells

Formaldehyde from 10 % stock was added to a 1 % final concentration to 10^7^ cells in 10 ml of SFX medium and samples were incubated in a rotator at room temperature for 15 min. The cross-linking was stopped by 0.125 M glycine. The samples were placed on ice and washed three times with PBS with 0.5 mM PMSF and centrifuged at 1000 rpm for 5 min at 4 °C. The pellet was resuspended in 10 ml of buffer I (25 mM HEPES pH 7.8, 1.5 mM MgCl2, 10 mM KCl, 0.1 % NP40, 1 mM DTT, 0.5 mM PMSF, Calbiochem Complete (EDTA)-free Protease Inhibitor Cocktail V) and placed on ice for 10 min. The suspension was homogenized in a Dounce homogenizer with pestle “B” 20 times and centrifuged at 2000 rpm for 5 min at 4 °C. The pellet was resuspended in 3 ml of buffer II (50 mM HEPES pH 7.8, 140 mM NaCl, 1 mM EDTA, 1 % Triton X-100, 0.1 % sodium deoxycholate, 0.1 % SDS, 0.5 mM PMSF, Calbiochem Complete (EDTA)-free Protease Inhibitor Cocktail V) and sonicated in a Bioruptor sonifier (40 alternating 30-s ON and 60-s OFF intervals). Finally, 50-μL aliquots were used to test the extent of sonication and to measure DNA concentration.

#### Pupae

A 500-mg pupa sample was ground in a mortar in liquid nitrogen and resuspended in 10 mL of buffer A (15 mM HEPES-KOH pH 7.6, 60 mM KCl, 15 mM NaCl, 13 mM EDTA, 0.1 mM EGTA, 0.15 mM spermine, 0.5 mM spermidine, 0.5 % NP-40, 0.5 mM DTT) supplemented with 0.5 mM PMSF and Calbiochem Protease Inhibitor Cocktail V. The suspension was then homogenized in a Dounce homogenizer with pestle “B” and filtered through Nylon Cell Strainer (BD Biosciences, USA). The homogenate was transferred to 3 mL of buffer A with 10 % sucrose (AS), and the nuclei were pelleted by centrifugation at 4000 g for 5 min at 4 °C. The pellet was resuspended in 5 mL of buffer A, homogenized again in a Dounce homogenizer, and transferred to 1.5 mL of buffer AS to collect the nuclei by centrifugation. The nuclear pellet was resuspended in wash buffer (15 mM HEPES-KOH pH 7.6, 60 mM KCl, 15 mM NaCl, 1 mM EDTA, 0.1 mM EGTA, 0.1 % NP-40, protease inhibitors) and cross-linked with 1 % formaldehyde for 15 min at room temperature. Cross-linking was stopped by adding glycine to a final concentration of 125 mM. The nuclei were washed with three 10-mL portions of wash buffer and resuspended in 1.5 mL of nuclear lysis buffer (15 mM HEPES pH 7.6, 140 mM NaCl, 1 mM EDTA, 0.1 mM EGTA, 1 % Triton X-100, 0.5 mM DTT, 0.1 % sodium deoxycholate, 0.1 % SDS, protease inhibitors). The suspension was sonicated in a Bioruptor sonifier (35 alternating 30-s ON and 60-s OFF intervals), and 50-μL aliquots were used to test the extent of sonication and to measure DNA concentration.

#### Immunoprecipitation

Debris was removed by centrifugation at 14,000 g for 10 min at 4 °C, and chromatin was pre-cleared with Protein A agarose (Pierce) blocked with BSA and salmon sperm DNA, with 50-μL aliquots of such pre-cleared chromatin being stored as input material. Samples containing 10–20 μg of DNA equivalent in 1 mL of nuclear lysis buffer were incubated overnight at 4 °C with rabbit antibodies against dCTCF (1:500) and CP190 (1:1000), mouse antibodies against FLAGx3 (1:200), or with non-specific IgG purified from rabbit or mouse pre-immune sera (control). Chromatin–antibody complexes were collected using blocked Protein A or G agarose at 4 °C over 5 h. After several rounds of washing with lysis buffer (as such and with 500 mM NaCl), LiCl buffer (20 mM Tris–HCl pH 8, 250 mM LiCl, 1 mM EDTA, 0.5 % NP-40, 0.5 % sodium deoxycholate, protease inhibitors), and TE buffer (10 mM Tris–HCl pH 8, 1 mM EDTA), the DNA was eluted with elution buffer (50 mM Tris–HCl, pH 8.0, 1 mM EDTA, 1 % SDS), the cross-links were reversed, and the precipitated DNA was extracted by the phenol–chloroform method. The enrichment of specific DNA fragments was analyzed by real-time PCR, using a StepOne Plus Thermal Cycler (Applied Biosystems). The primers used for PCR in ChIP experiments for genome fragments are shown in Additional file 9: Table S2.

## Additional files

Additional file 1: Figure S1. (A) Multiple sequence alignment of CTCF homologs in distant species. (B) Multiple sequence alignment of dCTCF proteins from different *Drosophila* species. (TIFF 15876 kb) Additional file 2: Figure S2. (A) Glutaraldehyde cross-linking of different dCTCF N-terminal derivatives as indicated (see also schematic in Fig. 1). Also included in panel A is glutaraldehyde cross-linking of the dCTCF-CTD sequence 612–818. (B) Limited proteolysis of the thioredoxin-fused dCTCF[1–205] protein with proteinase K or trypsin. Proteolysis-resistant fragments (indicated by the frame) were excised from the gel and subjected to MALDI-TOF mass spectrometry. Peptides found in these bands are indicated in the dCTCF N-terminal amino-acid sequence. Peptides recovered in the proteinase K digestion are underlined while peptides from trypsin digestion are shown in bold. (TIFF 4642 kb) Additional file 3: Table S1. The results of limited proteinase K or trypsin digestion. (DOC 36 kb) Additional file 4: Figure S3. Diagram showing the *dCTCF* gene and site of insertion of the *GE24185* transposon. Also included is the sequence of the wild-type *dCTCF* gene flanking the transposon insertion site and the sequences of the two excision derivatives *GEx52* and *GEx56*. (TIFF 2489 kb) Additional file 5: Figure S4. Abdominal transformations in females. Posterior sternites of wild type, *GE24185*, and *GE24185* females rescued with the *hsp83:dCTCF* ^*+*^, *hsp83:dCTCF* ^*ΔN*^, o*r hsp83:dCTCF* ^*ΔC*^ transgenes. Upper panel shows dark field images of the sternites A6 and A7, and lower panel shows the same sternites in bright field. The bristles on the A7 sternite of *GE24185* differ from wild type in that they are arranged in a manner that is characteristic of A6 (pointing outward rather than inward), and the trichome pattern of A7, evident in the dark field, likewise is characteristic of A6. This phenotypic transformation is rescued by the *hsp83:dCTCF* ^*+*^ and *hsp83:dCTCF* ^*ΔC*^ transgenes, but not by the *hsp83:dCTCF* ^*ΔN*^ transgene. (TIFF 3997 kb) Additional file 6: Figure S5. Histograms showing dCTCF occupancy in chromatin isolated from mid-late pupa at sequences corresponding to the BX-C insulators *Fab-3*, *Fab-4*, *Mcp*, *Fab-6*, *Fab-8*, the *Abd-D* promoter, and several other dCTCF insulators (9A1, 21E2, 24C4, 27B2, and 57B4R). Chromatin was prepared from 2–3 day old wild type (*WT*; *y* ^*1*^ *w* ^*1118*^ ) and homozyogous *GE24184* (*GE/GE*) mutant pupae. After fixation and processing, the isolated chromatin was incubated with antibodies directed against the C-terminal region of dCTCF. Sequences from tub, rpl32, and 62D regions were used as negative controls for dCTCF binding. The axis shows the scale for dCTCF enrichment. Error bars show standard deviations of quadruplicate PCR measurements in two biological replicates. The results are presented as a percentage of input DNA. (TIFF 2160 kb) Additional file 7: Figure S6. Western blots of protein extracts prepared from wild-type (*wt*; *y* ^*1*^ *w* ^*1118*^) and homozygous *GE24185* mutant pupae. The blots were probed with CP190, N-dCTCF, Pita antibodies. Protein levels in each extract were visualized by staining the membrane after protein transfer with Ponceau S. (TIFF 4911 kb) Additional file 8: Figure S7. Western blots of protein extracts from wild-type flies (*wt*; *y* ^*1*^ *w* ^*1*^) and homozygous *GE24185* flies carrying the *hsp83:dCTCF* ^*+*^, *hsp83:dCTCF* ^*ΔN*^, or *hsp83:dCTCF* ^*ΔC*^ *dCTCF* transgenes. All three of the transgene encoded proteins have an N-terminal 3xFLAG-tag and are detected with FLAG antibodies. The respective transgenes are indicated in the figure. (TIFF 668 kb) Additional file 9: Table S2. List of primers used. (DOC 51 kb)
