# Supplementary figures and images for: Functional role of dimerization and CP190 interacting domains of CTCF protein in Drosophila melanogaster
Source: BMC Biol. 2015 Aug 7;13:63. doi: 10.1186/s12915-015-0168-7 (PMC4528719; doi:10.1186/s12915-015-0168-7)

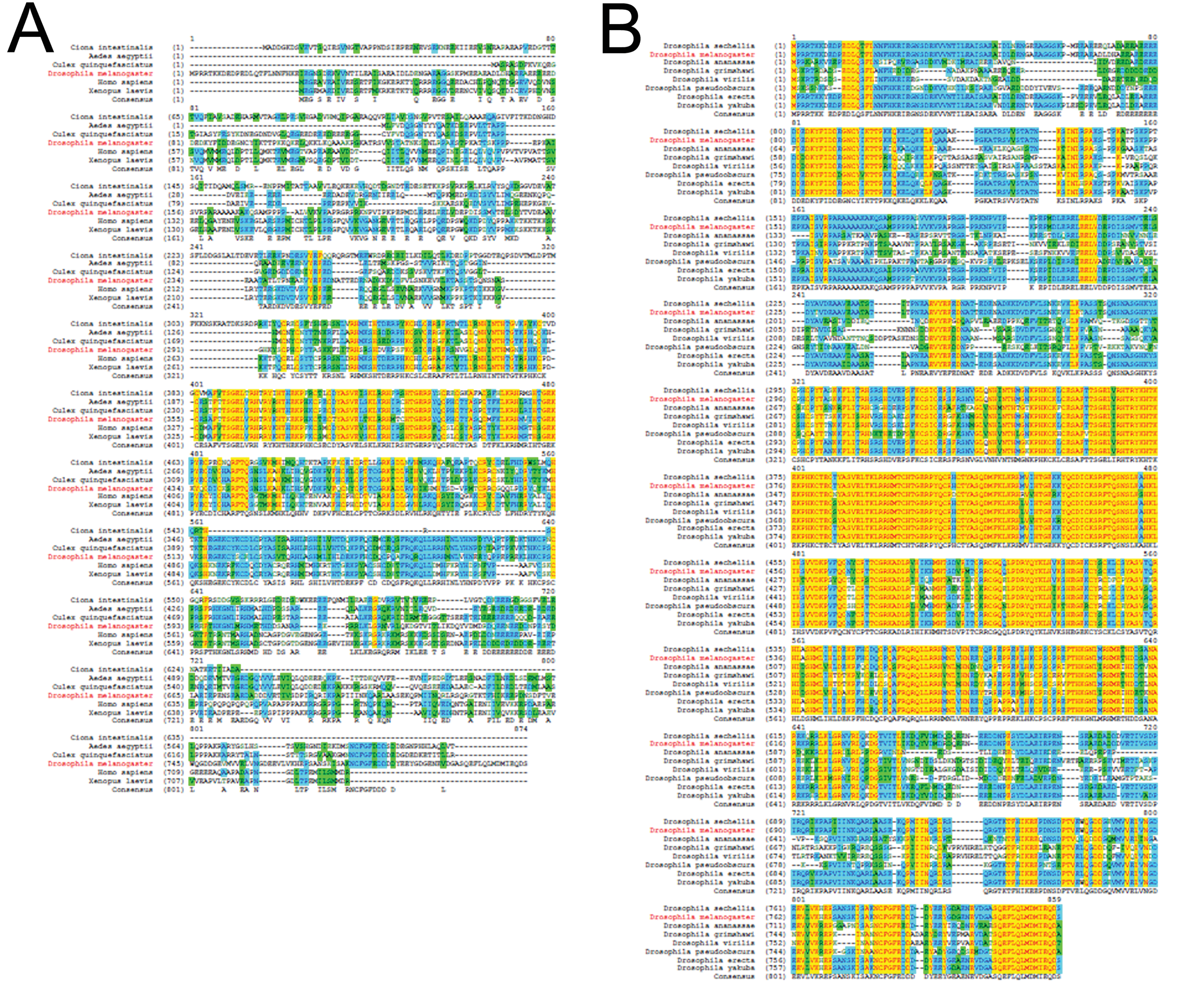

Supplement: Additional file 1: Figure S1. — (A) Multiple sequence alignment of CTCF homologs in distant species. (B) Multiple sequence alignment of dCTCF proteins from different Drosophila species. (TIFF 15876 kb) [file 12915_2015_168_MOESM1_ESM.tif]

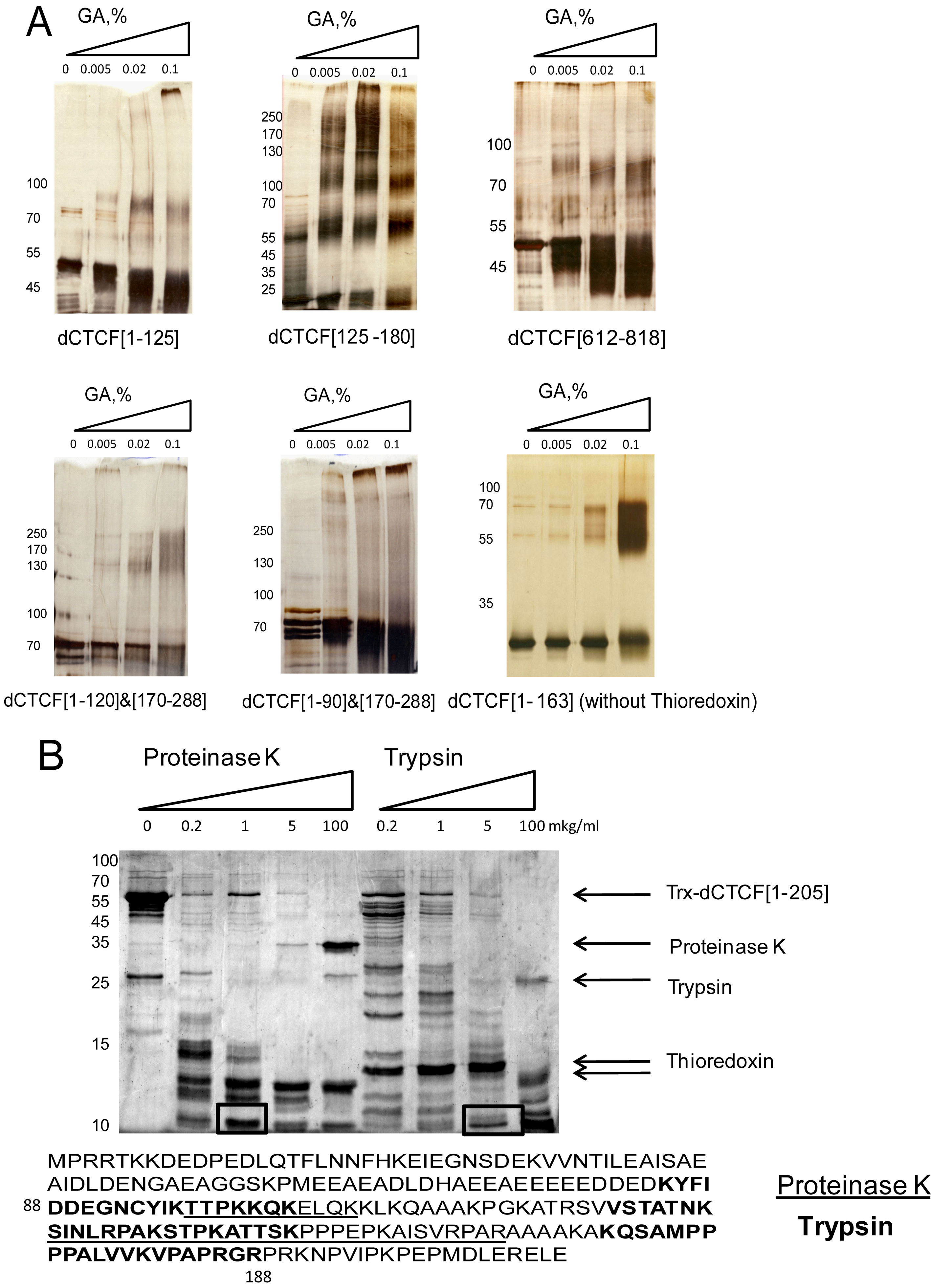

Supplement: Additional file 2: Figure S2. — (A) Glutaraldehyde cross-linking of different dCTCF N-terminal derivatives as indicated (see also schematic in Fig. 1). Also included in panel A is glutaraldehyde cross-linking of the dCTCF-CTD sequence 612–818. (B) Limited proteolysis of the thioredoxin-fused dCTCF[1–205] protein with proteinase K or trypsin. Proteolysis-resistant fragments (indicated by the frame) were excised from the gel and subjected to MALDI-TOF mass spectrometry. Peptides found in these bands are indicated in the dCTCF N-terminal amino-acid sequence. Peptides recovered in the proteinase K digestion are underlined while peptides from trypsin digestion are shown in bold. (TIFF 4642 kb) [file 12915_2015_168_MOESM2_ESM.tif]

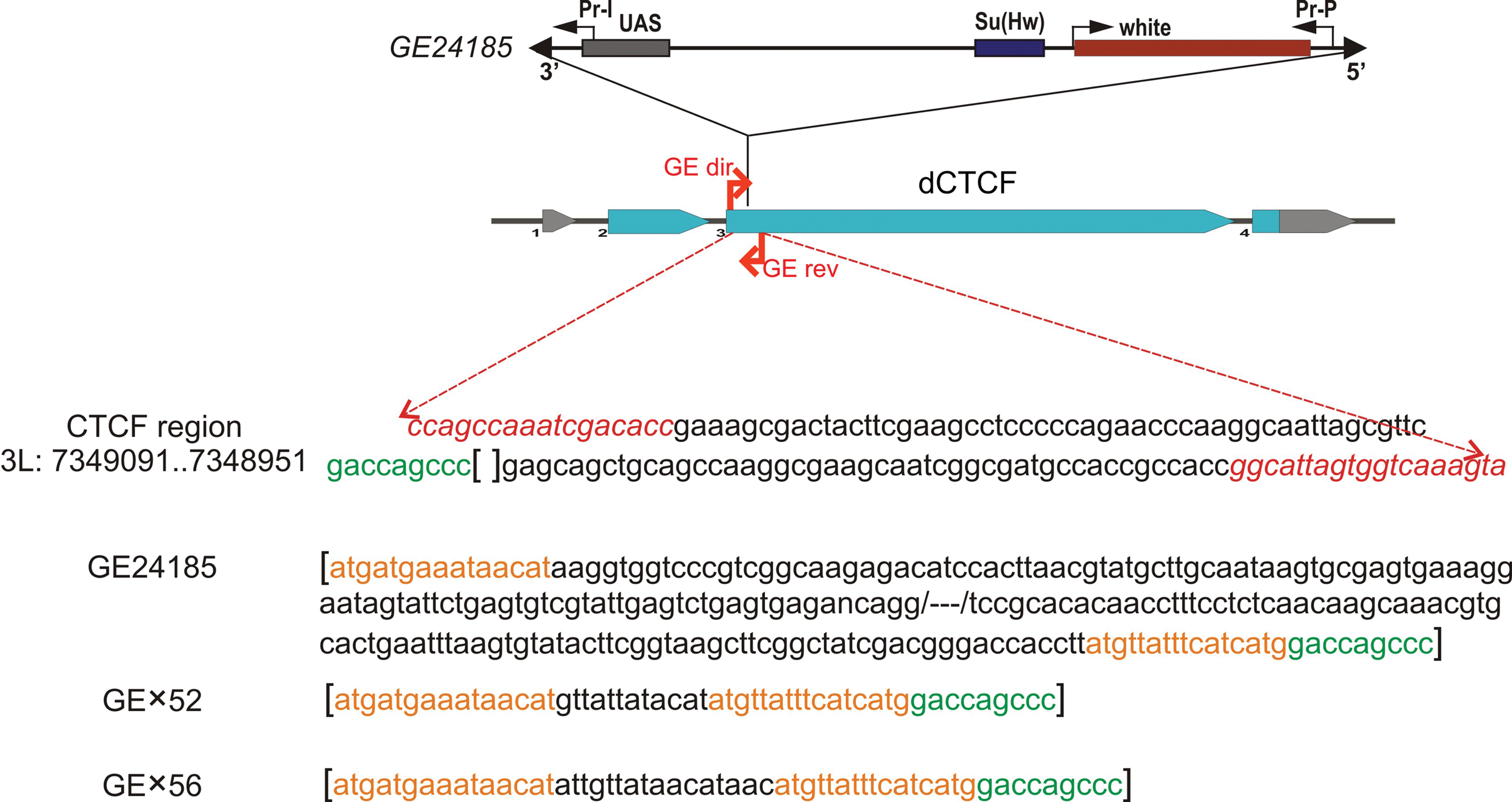

Supplement: Additional file 4: Figure S3. — Diagram showing the dCTCF gene and site of insertion of the GE24185 transposon. Also included is the sequence of the wild-type dCTCF gene flanking the transposon insertion site and the sequences of the two excision derivatives GEx52 and GEx56. (TIFF 2489 kb) [file 12915_2015_168_MOESM4_ESM.tif]

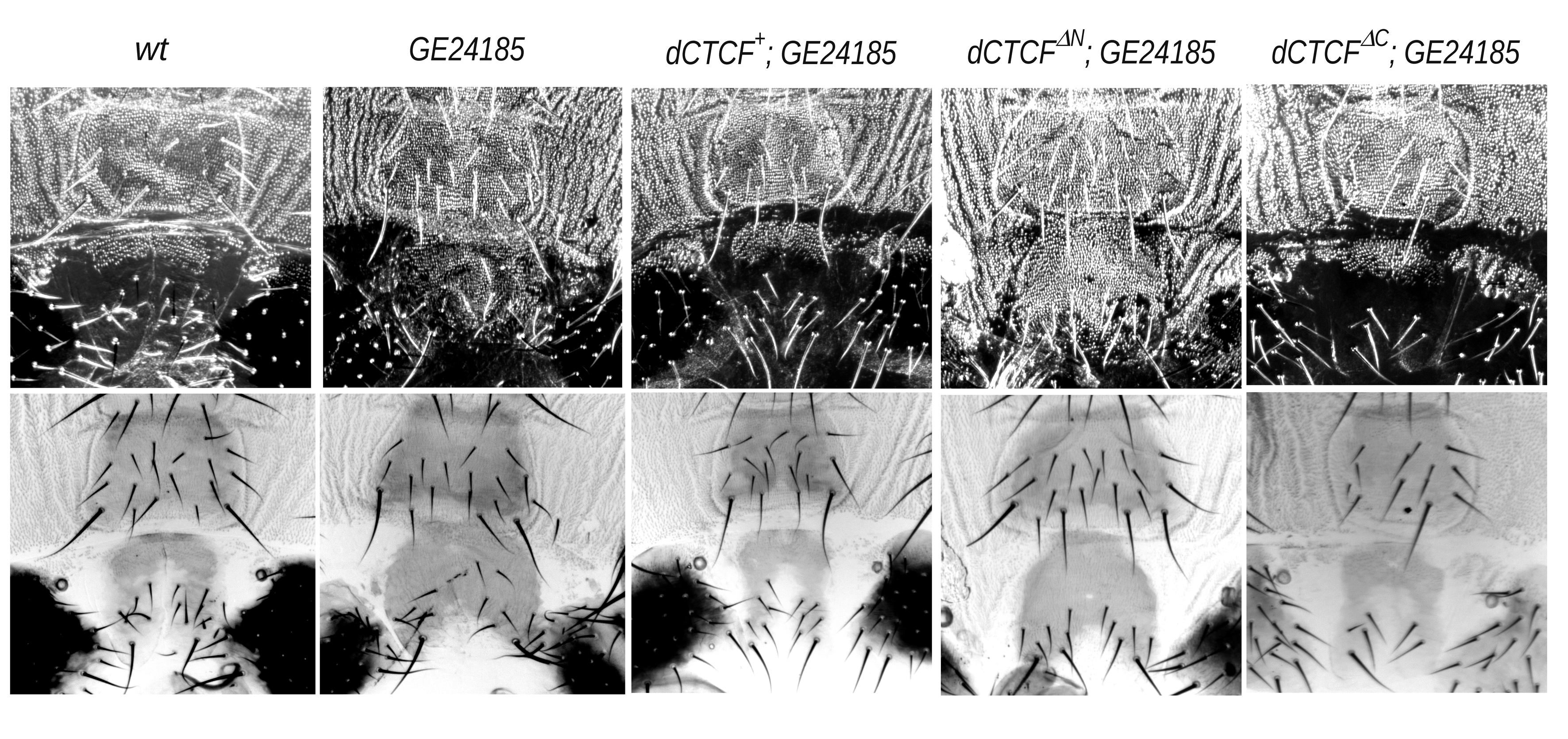

Supplement: Additional file 5: Figure S4. — Abdominal transformations in females. Posterior sternites of wild type, GE24185, and GE24185 females rescued with the hsp83:dCTCF +, hsp83:dCTCF ΔN, or hsp83:dCTCF ΔC transgenes. Upper panel shows dark field images of the sternites A6 and A7, and lower panel shows the same sternites in bright field. The bristles on the A7 sternite of GE24185 differ from wild type in that they are arranged in a manner that is characteristic of A6 (pointing outward rather than inward), and the trichome pattern of A7, evident in the dark field, likewise is characteristic of A6. This phenotypic transformation is rescued by the hsp83:dCTCF + and hsp83:dCTCF ΔC transgenes, but not by the hsp83:dCTCF ΔN transgene. (TIFF 3997 kb) [file 12915_2015_168_MOESM5_ESM.tif]

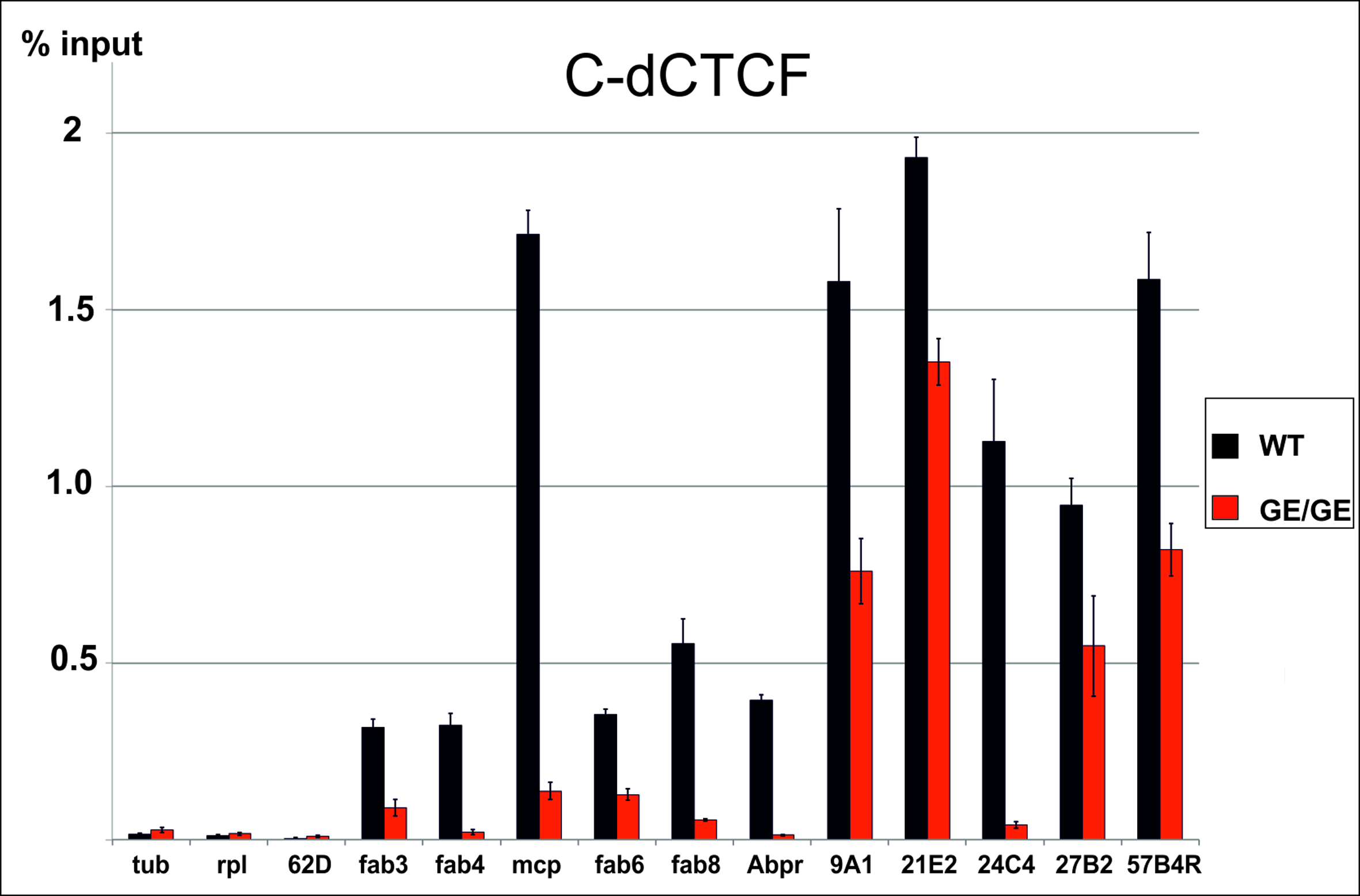

Supplement: Additional file 6: Figure S5. — Histograms showing dCTCF occupancy in chromatin isolated from mid-late pupa at sequences corresponding to the BX-C insulators Fab-3, Fab-4, Mcp, Fab-6, Fab-8, the Abd-D promoter, and several other dCTCF insulators (9A1, 21E2, 24C4, 27B2, and 57B4R). Chromatin was prepared from 2–3 day old wild type (WT; y 1 w 1118 ) and homozyogous GE24184 (GE/GE) mutant pupae. After fixation and processing, the isolated chromatin was incubated with antibodies directed against the C-terminal region of dCTCF. Sequences from tub, rpl32, and 62D regions were used as negative controls for dCTCF binding. The axis shows the scale for dCTCF enrichment. Error bars show standard deviations of quadruplicate PCR measurements in two biological replicates. The results are presented as a percentage of input DNA. (TIFF 2160 kb) [file 12915_2015_168_MOESM6_ESM.tif]

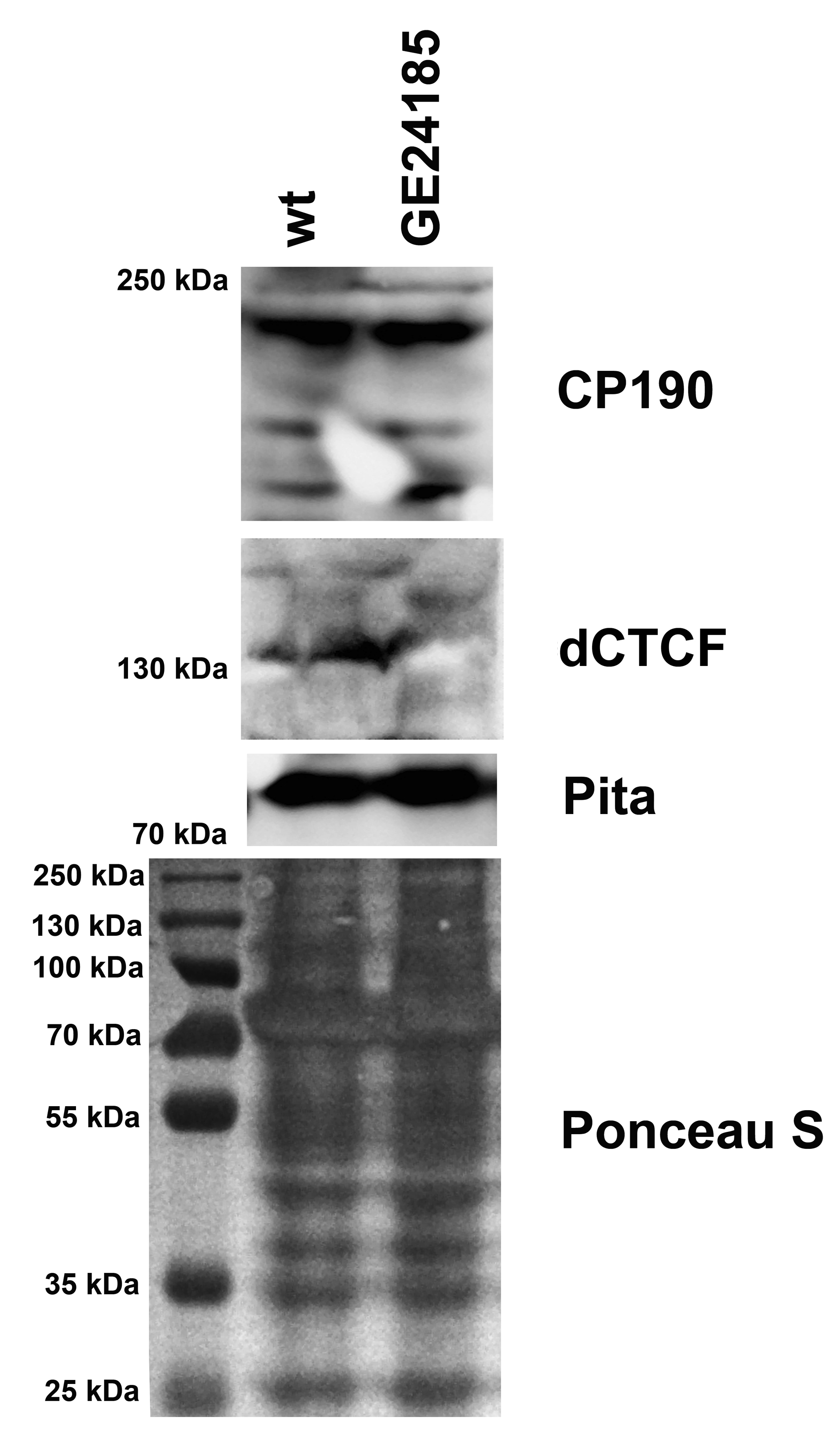

Supplement: Additional file 7: Figure S6. — Western blots of protein extracts prepared from wild-type (wt; y 1 w 1118) and homozygous GE24185 mutant pupae. The blots were probed with CP190, N-dCTCF, Pita antibodies. Protein levels in each extract were visualized by staining the membrane after protein transfer with Ponceau S. (TIFF 4911 kb) [file 12915_2015_168_MOESM7_ESM.tif]

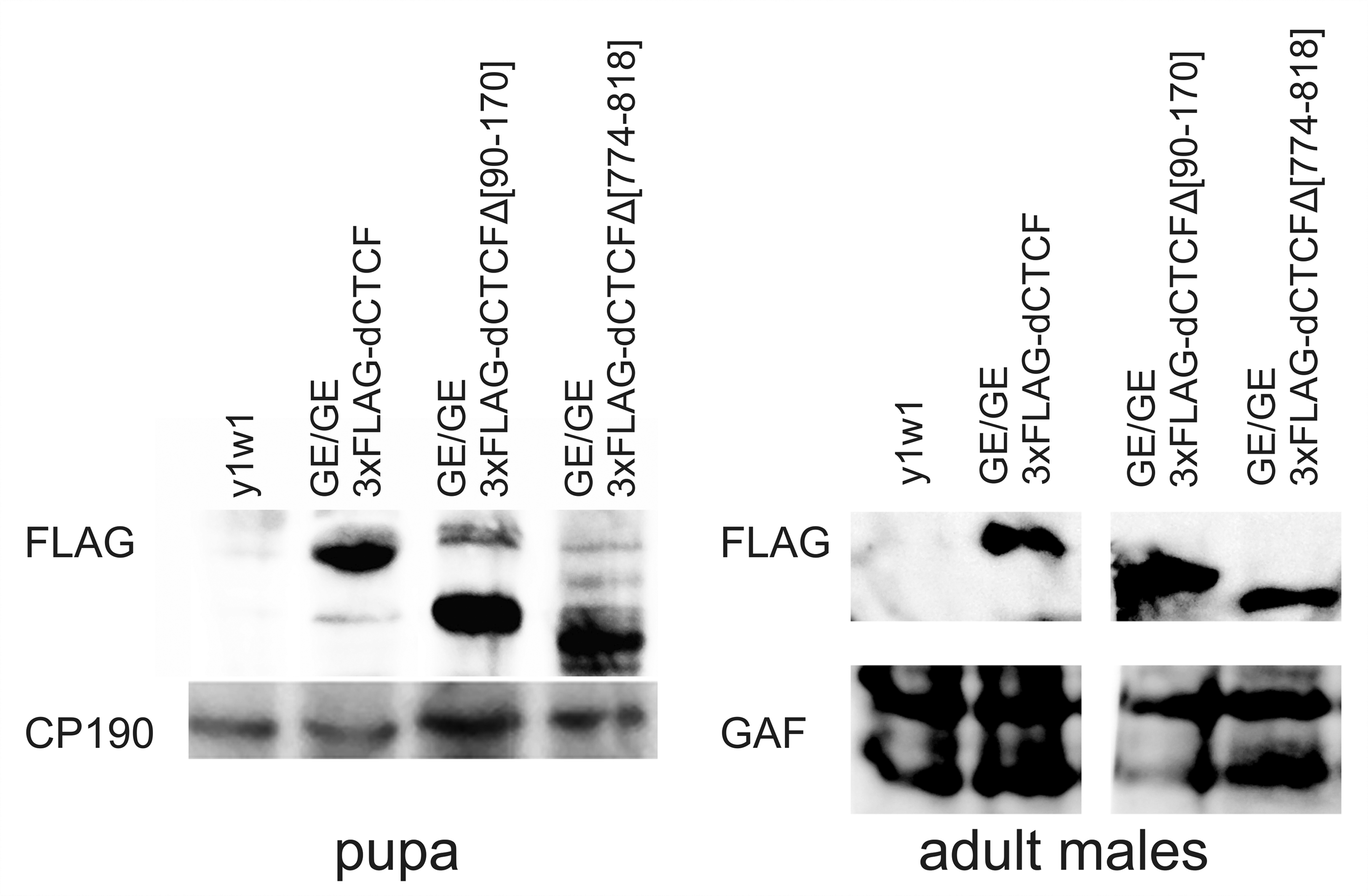

Supplement: Additional file 8: Figure S7. — Western blots of protein extracts from wild-type flies (wt; y 1 w 1) and homozygous GE24185 flies carrying the hsp83:dCTCF +, hsp83:dCTCF ΔN, or hsp83:dCTCF ΔC dCTCF transgenes. All three of the transgene encoded proteins have an N-terminal 3xFLAG-tag and are detected with FLAG antibodies. The respective transgenes are indicated in the figure. (TIFF 668 kb) [file 12915_2015_168_MOESM8_ESM.tif]
