# Supplementary material for: Functional role of dimerization and CP190 interacting domains of CTCF protein in Drosophila melanogaster
Source: BMC Biol. 2015 Aug 7;13:63. doi: 10.1186/s12915-015-0168-7 (PMC4528719; doi:10.1186/s12915-015-0168-7)
Supplement: Additional file 3: Table S1. — The results of limited proteinase K or trypsin digestion. (DOC 36 kb) [file 12915_2015_168_MOESM3_ESM.doc]

| Peptide mass, Da | Residues | Sequence |
| --- | --- | --- |
| **Proteinase K band** | | |
| 869,533 | 154-161 | AISVRPAR |
| 898,554 | 131-138 | SINLRPAK |
| 1152,63 | 143-153 | ATTSKPPPEPK |
| 1328,71 | 97-107 | TTPKKQKELQK |
| 1565,83 | 139-153 | STPKATTSKPPPEPK |
| Masses not identified: 797.36; 1791.88 | | |
| **Trypsin band** | | |
| 2196,19 | 168-188 | KQSAMPPPPALVVKVPAPRGR |
| 2418,28 | 84-103 | KYFIDDEGNCYIKTTPKKQK |
| 2501,34 | 124-147 | VSTATNKSINLRPAKSTPKATTSK |
| Masses not identified 684.21; 714.96; 1320.60; 1407.70; 1531.77; 1838.92; 1867.93; 2023.12; 2136.11; 2184.19 | | |
| **Band identified as Thioredoxin (in Trypsin digestion)** | | |
| 1001,65 | 75- 83 | GIPTLLLFK |
| 1267,67 | 59- 70 | LNIDQNPGTAPK |
| 1400,69 | 118-129 | HHHHHSSGLVPR |
| 1731,85 | 5-19 | IIHLTDDSFDTDVLK |
| 2062,02 | 2-19 | SDKIIHLTDDSFDTDVLK |
| 2249,06 | 140-158 | KFERQHMDSPDLGTDDDDK |
| Masses not identified: 1041.62; 1096.67; 1154.73; 1214.68; 1333.72; 1549.86; 1790.90. | | |
