# Supplementary material for: Functional role of dimerization and CP190 interacting domains of CTCF protein in Drosophila melanogaster
Source: BMC Biol. 2015 Aug 7;13:63. doi: 10.1186/s12915-015-0168-7 (PMC4528719; doi:10.1186/s12915-015-0168-7)
Supplement: Additional file 9: Table S2. — List of primers used. (DOC 51 kb) [file 12915_2015_168_MOESM9_ESM.doc]

| **Primer** | **Sequence (5'---->3')** |
| --- | --- |
| ***Preparation of constructs*** | |
| dCTCFdir | ggatccatgccaaggaggaca |
| dCTCF90r | caactagtctcgtcatctatgaaatactta |
| dCTCF120r | ggaactagtagctttccccggct |
| dCTCF170d | gactagtgcgatgccaccgcca |
| dCTCF611d | gatggtaccgcgaatgctcgcgaaaag |
| dCTCF610r | cactagtatcgtgagtctccatgtg |
| dCTCF723d | agaactagtcggggcaccaagacg |
| dCTCF774r | ccaagcttcgagggcccgctcggttcgtgctt |
| ***CHIP analysis of pupa*** | |
| tub RT dir | gctttcccaagaagctcataca |
| tub RT rev | ggttcagtgcggtattatccag |
| rpl32_RT_d | gttcgatccgtaaccgatgt |
| rpl32_RT_r | ccagtcggatcgatatgctaa |
| 62D_d | tgataccaggcgaacagaaatc |
| 62D_r | tttgggcttggtgagaacag |
| FAB3_cts_d | taaaggccaatgcacaaaggcgac |
| FAB3_cts_r | acgcttcagcgaacggaatacaga |
| FAB4_cts_d | caatttgccaatatttcgcagtccct |
| FAB4_cts_r | ccctggcgggcatatgagaaa |
| MCP_cts_d | aaagtcgggtctgcaaataagg |
| MCP_cts_r | gcataagctgcaaaagaaaaacaa |
| FAB6_cts_d | agctaaacccgatttgctttgccg |
| FAB6_cts_r | ctgcccagtgggagatacaaagat |
| FAB8_cts_d | tgttggtgagcaagcgaaga |
| FAB8_cts_r | cgaacattttttacgcgacatgt |
| Abpr_cts_d | ccaacaacaagccaactaactaca |
| Abpr_cts_r | acgaacaaaaaacgctctcagact |
| 9A1_cts_d | ttatgcccgcacaacaatta |
| 9A1_cts_r | gaaatgggcgtcgtagctta |
| 21E2_cts_d | ccatctgttgttccctgaaag |
| 21E2_cts_r | gctgttaattccagtgtgtgta |
| 24C4_cts_d | caagatgcacgacacaaataag |
| 24C4_cts_r | ccataaagacagccctcaac |
| 27B2_cts_d | ggtcagcagaaacacagtta |
| 27B2_cts_r | caacaacaacagcaacagatta |
| 57B4R_cts_d | acacattacaaagtgtttgcc |
| 57B4R_cts_r | ccaccgcgttaagttctac |
